# The Phylogeny and Biogeographic History of Ashes (*Fraxinus*, Oleaceae) Highlight the Roles of Migration and Vicariance in the Diversification of Temperate Trees

**DOI:** 10.1371/journal.pone.0080431

**Published:** 2013-11-21

**Authors:** Damien Daniel Hinsinger, Jolly Basak, Myriam Gaudeul, Corinne Cruaud, Paola Bertolino, Nathalie Frascaria-Lacoste, Jean Bousquet

**Affiliations:** 1 Laboratoire Ecologie, Systématique et Evolution, Unité mixte de recherche 8079, AgroParisTech, Orsay, France; 2 Laboratoire Ecologie, Systématique et Evolution, Unité mixte de recherche 8079, Centre national de la recherche scientifique, Orsay, France; 3 Laboratoire Ecologie, Systématique et Evolution, Unité mixte de recherche 8079, Université Paris Sud, Orsay, France; 4 Canada Research Chair in Forest and Environmental Genomics, Centre for Forest Research and Institute for Systems and Integrative Biology, Université Laval, Québec, Québec, Canada; 5 Unité mixte de recherche 7205 ‘Origine, Structure et Evolution de la Biodiversité’, Muséum National d'Histoire Naturelle, Paris, France; 6 Genoscope, Centre National de Séquençage, Evry, France; Bangor University, United Kingdom

## Abstract

The cosmopolitan genus *Fraxinus*, which comprises about 40 species of temperate trees and shrubs occupying various habitats in the Northern Hemisphere, represents a useful model to study speciation in long-lived angiosperms. We used nuclear external transcribed spacers (nETS), *phantastica* gene sequences, and two chloroplast loci (*trnH*-*psbA* and *rpl32*-*trnL*) in combination with previously published and newly obtained nITS sequences to produce a time-calibrated multi-locus phylogeny of the genus. We then inferred the biogeographic history and evolution of floral morphology. An early dispersal event could be inferred from North America to Asia during the Oligocene, leading to the diversification of the section *Melioides sensus lato*. Another intercontinental dispersal originating from the Eurasian section of *Fraxinus* could be dated from the Miocene and resulted in the speciation of *F. nigra* in North America. In addition, vicariance was inferred to account for the distribution of the other Old World species (sections *Sciadanthus*, *Fraxinus* and *Ornus*). Geographic speciation likely involving dispersal and vicariance could also be inferred from the phylogenetic grouping of geographically close taxa. Molecular dating suggested that the initial divergence of the taxonomical sections occurred during the middle and late Eocene and Oligocene periods, whereas diversification within sections occurred mostly during the late Oligocene and Miocene, which is consistent with the climate warming and accompanying large distributional changes observed during these periods. These various results underline the importance of dispersal and vicariance in promoting geographic speciation and diversification in *Fraxinus*. Similarities in life history, reproductive and demographic attributes as well as geographical distribution patterns suggest that many other temperate trees should exhibit similar speciation patterns. On the other hand, the observed parallel evolution and reversions in floral morphology would imply a major influence of environmental pressure. The phylogeny obtained and its biogeographical implications should facilitate future studies on the evolution of complex adaptive characters, such as habitat preference, and their possible roles in promoting divergent evolution in trees.

## Introduction

Vicariance and isolation have been suggested as important drivers of diversification in trees, especially for those distributed over wide areas that have previously been subjected to fragmentation, or experienced major distributional changes (e.g. [1]). Colonization of diverse habitats could also promote divergent evolution. For example, divergent evolution could be associated with transitions from insect to wind pollination, which could lead to simpler flower morphology [2]. A change in pollination mode might also affect the genetic structure *via* changes in effective population size [3] and the rate of speciation.

Members of the genus *Fraxinus*, known as ash trees, comprise 43 recognized species [4], and are widely distributed in the northern temperate zone from Eurasia to North America. They display a range of morphologies (e.g., shrubs to trees; simple to compound leaves) and are also found in diverse habitats, from semi-deserts to subtropical or temperate environments, and from sea level to subalpine altitudes. *Fraxinus* shows great diversity in floral morphology, with taxa with complete, incomplete and naked flowers (i.e., no corolla or calyx), with two or four petals, and in the case of *F. excelsior*, with male, female or hermaphrodite flowers on the same tree [5]. Given this diversity of locations, habitats, and morphologies, the cosmopolitan *Fraxinus* should represent an informative model to investigate speciation patterns in long-lived angiosperm species in relation to divergent evolution caused by vicariance or adaptation to different habitats.

Evolutionary inferences are valuable only if conducted in the proper phylogenetic context and in the light of a sound knowledge of the biogeographic history [6], [7]. Several studies have investigated the phylogeny of *Fraxinus* using nuclear internal transcribed spacer (nITS) [4], [8] and chlroroplastic DNA (cpDNA) sequences [4], [9]. The monophyly of this genus in the tribe Oleeae has been confirmed [10] and six sections (*Dipetaleae*, *Fraxinus*, *Melioides*, *Ornus*, *Pauciflorae*, and *Sciadanthus*) have been delimited on the basis of molecular (reciprocal monophyly) and morphological characters (flower and samara morphology) [4] (Table 1). However, three *Fraxinus* species (*F. cuspidata*, *F. spaethiana*, and *F. chiisanensis*) remained unclassified (*incertae sedis*) [4]. The species in the different sections usually form cohesive continental groups (the sections *Dipetaleae*, *Melioides*, and *Pauciflorae* in North America; and the sections *Fraxinus*, *Ornus*, and *Sciadanthus* in Eurasia).

**Table 1:** Different classification schemes of the genus *Fraxinus*, including the revisions proposed in this study, and geographic distributions of species.

Sections based on [8]	Sections based on [4]	Sections based on this study	Species	Synonyms used in this study	Distribution
***Ornus***	***Ornus***	***Ornus***	*ornus* L.		Mediterranean area, N Africa and SW Asia
***Ornaster***			*apertisquamifera* Hara		Japan
			*bungeana* DC		China
			*floribunda* Wall.	*retusa* Champ. ex Benth.var. *henryana*	C & E Asia (from Afghanistan to Japan)
			*griffithii* G. B. Clarke		E Asia (from NE India to Japan and Indonesia)
			*lanuginosa* Koidz. var. *lanuginosa* and var. *serrata* (Nakai) Hara		Japan
			*malacophylla* Hemsl.		China, Thailand
			*paxiana* Lingelsh.	*sikkimensis* (Lingelsh.) Handel-Mazzetti	Himalayas, China
			*raibocarpa* Regel		C Asia (Turkestan mountains, Iran, Pakistan, Afghanistan)
			*sieboldiana* Blume	*mariesii* Hook. f.	China, Japan, Korea
			*trifoliolata* W. W. Smith		China
			*baroniana* Diels		China
			*chinensis* Roxb.		China, Japan, Korea, Vietnam
			*longicuspis* Sieb. & Zucc.		Japan
			*micrantha* Lingelsh.		C Asia (Punjab to Nepal, Himalayas)
***Bumelioides***	***Dipetalae***	***Dipetalae***	*anomala* Torr. ex S. Wats.		SW USA, N Mexico
			*dipetala* Hook. et Arn.	*trifoliata* (Torr.) Lewis & Epling	SW USA, N Mexico (Baja California)
			*quadrangulata* Michx.		E & C USA, C Canada
	***Fraxinus***	***Fraxinus***	*angustifolia* Vahl. ssp. *angustifolia*	*monophylla* Desf.	SW Europe
			*angustifolia* Vahl. ssp. *oxycarpa* (M.Bieb. ex Willd.) Franco & Rocha Afonso	*oxycarpa* Willd., *pallisiae* A. J. Willmott, *obliqua* Tausch	SE Europe
			*angustifolia* Vahl. ssp. *syriaca* (Boiss.) Yalt.	*potamophila* Herder, *holotricha* Koehne, *sogdiana* Bunge, s*yriaca* Boiss.	W & E Asia (Turkey to Pakistan and Russia) and Algeria
			*excelsior* L.	*turkestanica* Carrière	C & N Europe
			*mandshurica* Rupr.		E Asia (China, Japan, Korea, E Russia)
			*nigra* Marsh.		E USA, E Canada
		***Melioides***	*platypoda* Oliv.		E Asia
***Melioides***	***Melioides***		*americana* L.	*biltmoreana* Beadle	E USA, E Canada
			*berlandieriana* A. DC.		SW USA, NE Mexico
			*caroliniana* Mill.		SE USA
			*latifolia* Benth.		W USA
			*papillosa* Lingelsh.		SW USA (SE Arizona, SW New Mexico, Texas), Mexico (W Chihuahua, NE Sonora)
			*pennsylvanica* Marsh.	*richardii* Bosc	C & E USA, Canada
			*profunda* (Bush) Bush	*tomentosa* Michx. f.	E USA
			*texensis* A. Gray		S USA (Texas)
			*uhdei* (Wenzig) Lingelsh.		Guatemala, Honduras, Mexico, USA (Hawaii, Puerto Rico)
			*velutina* Torr		SW USA, N Mexico
***Dipetalae***	***incertae sedis***		*cuspidata* Torr.		SW USA, Mexico
**N.A.**			*chiisanensis* Nakai		Korea
			*spaethiana* Lingelsh.		Japan
	***Pauciflorae***	***Pauciflorae***	*dubia* (Willd. ex Schult & Schult f.) P. S. Green & M. Nee		Mexico, Guatemala
			*gooddingii* Little		SW USA (Arizona), N Mexico
			*greggii* A. Gray		SW USA, Mexico
			*purpusii* Brandegee		Mexico, Guatemala
			*rufescens* Lingelsh.		Mexico
	***Sciadanthus***	***Sciadanthus***	*xanthoxyloides* (G. Don) DC.		N Africa (Algeria, Morocco) to Asia (Afghanistan to China)
			*hubeiensis* S. Z. Qu, C. B. Shang & P. L. Su	*hoopiensis*	China

N.A.: not available, taxa that were not included in the considered study.

In addition to cases of incongruence, previous phylogenetic studies failed to achieve a complete resolution of relationships at the species level within the *Fraxinus* genus and suggested that some morphological species may not be monophyletic [4], [8]. These findings are suggestive of incomplete lineage sorting [11], [12] or reticulate evolution [13], [14], which could make phylogenetic inferences difficult. The occurrence of natural hybridization between ash taxa in parapatric or sympatric contact [15], [16] may add to this difficulty and illustrates that reproductive isolation is a slow process that may affect speciation patterns and speed in *Fraxinus*, as noted for other temperate and boreal trees (e.g. [13], [17]).

The evolution of floral morphology has been investigated in *Fraxinus* by mapping floral characters onto phylogenies. A trend toward sequential simplification of floral structure associated with the loss of corolla and calyx was reported, but a reversal of this was also observed [8]. The same trend toward simplification as well as reversal was also inferred using a more complete phylogeny [4], but there were conflicting findings between these two studies owing to incongruences between the estimated phylogenies. Against this background, improving the resolution of the phylogeny of *Fraxinus* by considering more loci should improve our understanding of the evolution of floral morphology by enabling better estimates of the frequencies of reversals or multiple losses of floral components, and how these floral changes are linked to the biogeographic and speciation history, as well as the colonization of new habitats.

The objectives of this study were to reconstruct the complete phylogeny of *Fraxinus* from multiple loci for an accurate assessment of the relationships among the sections of the genus, infer the biogeographic history and speciation patterns of this cosmopolitan angiosperm group, and better understand the long-term drivers of speciation in temperate tree taxa. A more robust phylogeny should also improve our understanding of the evolution of phenotypic characters such as floral morphology, and facilitate the distinction between parallel and convergent evolution from a common ancestry. An ancillary objective was to compare the phylogenies obtained from structurally different loci (the repeated nuclear ribosomal regions, cpDNA intergenic regions, and a low-copy nuclear gene) in order to detect possible sources of locus-specific conflicts that could blur phylogenetic inference.

## Materials and Methods

### Plant material

We collected 292 samples (1 to 28 individuals per species) for 40 *Fraxinus* species (from a total of 43 *sensu* [4]) that represent almost the entire genus, from arboreta (n = 198), herbaria (n = 17) or the field (n = 87) (see Table S1 for fieldwork information and vouchers). The sampling did not require any specific permits, given that it was carried out on government-owned sites and did not involve endangered or protected species. In the present study, we retained the classification and synonymies of Wallander [4] because of her exhaustive sampling of the genus (Table 1). Outgroups from the Oleaceae were selected in accordance with Wallander and Albert [10] (*Forsythia*×*intermedia*, *Jasminum* sp., *Ligustrum vulgare*, *Olea europaea*, *Osmanthus fragrans*, *Phillyrea angustifolia*, and *Syringa vulgaris*; see Table S1 for details).

The morphological identification of *Fraxinus* samples can be problematic if the samples are from an area of sympatry in which interspecific hybridization may occur, or because of misidentification due to mislabelling in arboreta or herbaria, or synonymy of taxa. To overcome these issues and render estimated phylogenies more robust, we avoided sampling in areas of sympatry or parapatry, used multiple individuals, and sampled from several sources (including the field, herbaria, and arboreta) (Table S1). Indeed, we expected that, if hybridization or misidentification occurred, only one or a few individuals in a particular species would be affected; as a consequence, most individuals would still be classified in a monophyletic group representative of the species (see Results). Most specimens have been deposited as herbarium vouchers for further morphological confirmation of their identity (see Table S1).

For fresh samples, 50 mg of fresh leaves were dehydrated in an alcohol/acetone 70∶30 solution and stored dry, following a modified version of the protocol of Fernandez-Manjarres et al. [15]. Extraction of DNA was performed for all samples using the DNeasy Plant Mini Kit (Qiagen, Valencia, California, USA) following the manufacturer's instructions.

### Sequence datasets

To reconstruct the phylogeny of *Fraxinus*, we used two cpDNA intergenic regions previously used in *Fraxinus* (*trnH-psbA* and *rpl32-trnL*) [18], and also sequenced the nuclear external transcribed spacer (nETS) regions and the nuclear low-copy gene *phantastica*. In addition, newly sampled individuals were sequenced for the nuclear ribosomal nITS and added to those already available in GenBank. Of these five loci, three have been used previously in molecular studies of *Fraxinus*. Sequences of the *trnH-psbA* and *rpl32-trnL* loci were tested recently as part of a possible DNA barcoding system for the genus *Fraxinus* [18], whereas nITS sequences were used in previous phylogenetic analyses of this genus [4], [8].

#### Chloroplast sequences

Both *trnH-psbA* and *rpl32-trnL* sequences were obtained from Arca et al. [18] and concatenated to construct a chloroplast dataset.

#### Nuclear ribosomal spacers (nETS and nITS)

DNA was amplified from *Fraxinus* samples using the SST-ETS (GGC WTG TKT GGG TAT GTT GGA T) and 18S-ETS (ACT TAC ACA TGC ATG GCT TAA TCT) primers previously used in *Syringa* (Oleaceae) [19]. For some samples for which amplification using this primer set was difficult, the primers ETS-F1 (ATG CCT GTT CAT TGG ATG) and ETS-R1 (AAG YCC AVV ACR AGG ARG) were designed *de novo*. The PCR reaction mix contained two units of *Taq* DNA polymerase MP-Biomedicals®, 1× buffer, 1.5 mM MgCl_2_, 0.25 mM dNTPs, 1 µM each primer, 0.1 mg/ml DMSO, and 30 ng of template DNA in a final volume of 25 µl. All reactions were carried out under the following thermal cycle program: 2 min at 94°C, 5 cycles of 15 s at 94°C, 30 s at 57°C, with a touch-down of −1°C per cycle, and 30 s at 72°C, followed by 50 cycles of 15 s at 94°C, 30 s at 52°C, and 30 s at 72°C, and finally 7 min at 72°C. Reactions were performed in a Mastercycler® ep gradient S (Eppendorf, Hamburg, Germany). The PCR quality was checked on 1.5% agarose gels and the amplified PCR products were stored at 4°C.

In addition to Genbank sequences from Wallander [4], we generated nITS sequences for 127 individuals using the primers ITS4 and ITS5 [20], [21], in accordance with the reaction protocol from the same study. For samples for which amplification using either of these primer sets was more difficult, nITS1 and nITS2 were amplified separately using the primer pairs ITS1/ITS6 and ITS7/ITS8 [8]. The PCR quality was checked on 1.5% agarose gels and the amplified PCR products were stored at 4°C until direct sequencing. For nITS, sequences from Genbank and new sequences were combined in a “nITS dataset” that included 43 species.

#### The phantastica gene

The *phantastica* gene is a single-copy gene that encodes a transcription factor involved in leaf morphology, and the growth and dorsoventrality of the lateral organs in *Antirrhinum* [22]. It has also been shown to determine leaflet placement in compound leaves of various angiosperms, notably *F. americana* [23]. It was retained for use in the present study after the preliminary sequencing of several nuclear regions (e.g. *floricaula*, *frigida, phantastica*, *adh*, *waxy*, *chi*, *tpi*, *pgi*, *waxy*, and *g3pdh*), which revealed that the *phantastica* gene is sufficiently conserved to be realiably aligned across the tribe Oleeae of Oleaceae, while being sufficiently variable to resolve relationships in the genus *Fraxinus* (data not shown). Moreover, very few heterozygote positions were identified, none of which revealed evidence of recombination. To our knowledge, this is the first time that *phantastica* has been used for phylogenetic inference.

The Phan-F-M13 (TGTAAAACGACGGCCAGGGGTAAGTGGTGGGAGGTTT) and Phan-R-M13 (CAG GAAACAGCTATGACCCGACTCGAGTTGCTGTTC) primers (Basak J., Visva-Bharati Univ., India, unpublished data) were used to amplify an approximately 625-bp-long fragment in the exon. The PCR reaction mix contained two units of *Taq* DNA polymerase MP-Biomedicals®, 1× buffer, 1.5 mM MgCl_2_, 0.25 mM dNTPs, 1 µM each primer, 0.1 mg/ml DMSO, and 30 ng of template DNA for a final volume of 25 µl. All reactions involved the following thermal cycle program: 2 min at 94°C, 5 cycles of 15 s at 94°C, 30 s at 62°C, with a touch-down of −1°C per cycle, and 30 s at 72°C, followed by 50 cycles of 15 s at 94°C, 30 s at 57°C, and 30 s at 72°C, and finally 7 min at 72°C. Reactions were performed in a Mastercycler® ep gradient S (Eppendorf, Hamburg, Germany). The PCR quality was checked on 1.5% agarose gels and the amplified PCR products were stored at 4°C until direct sequencing.

### DNA sequencing and analyses

All sequencing was carried out at the Génoscope facility (Centre National de Séquençage, Evry, France). The PCR products were purified using exonuclease I and phosphatase, and sequenced using the BigDyeTerminator V3.1 kit (Applied Biosystems) and an ABI 3730XL sequencer. All regions were sequenced for both DNA strands to confirm the accuracy of the sequences. The standard M13 primers [24] were used for *phantastica* sequencing, as described by Ivanova et al. [25]. All new sequences have been deposited in Genbank under Accession numbers HQ705327 to HQ705604 (nETS), HQ705200 to HQ705326 (nITS), and HM242423 to HM242846 (*phantastica*).

Sequence alignment and contigs were checked using CodonCode Aligner version 1.6.3 (Codon Code Corporation), and sequences were aligned manually using using Muscle [26] and Se-Al [27]. The datasets (cpDNA, nITS, nETS, and *phantastica*) were analysed separately, and then concatenated to conduct a “total evidence” analysis. Aligned sequences were used to construct phylogenetic trees based on the maximum likelihood (ML) criterion and Bayesian inference method (BIM). All ML trees were constructed using PhyML [28]. Indels were not considered. Using MrModeltest 2.3 [29], we chose the GTR+I+G model under the AIC criterion, which provides a better estimate of branch lengths than more complex models [30] and does not influence the probabilities of the nodes, even in over-parameterized analysis [31]. We included all individuals, which resulted in a total of 302 samples sequenced for at least one locus (including the outgroups). Bootstrap values were estimated with 1,000 pseudo-replicates.

Regarding the nearly identical nITS sequences reported for *F. platypoda* and *F. mandshurica* [4], and given that the two species are in sympatry in Japan, it is possible that these individuals were misidentified and were from the same species, most likely *F. mandshurica*. To avoid such potential problems related to synonymy, we used many (n = 10) individuals sampled from natural populations in Japan. They all formed a monophyletic group (with the arboretum specimens) for all the different datasets and methods of phylogenetic reconstruction used in the present study (see Figures S2, S3, S4).

All BIM analyses were performed on a reduced dataset using MrBayes [32]. This reduced dataset was created by using one individual per species following Wallander [4], with the selection of individuals located within the ML tree at positions that were reasonable considering their identity for each data partition, that is, grouping with other individuals from the same species. Doing otherwise would have resulted in the need to consider the entire 302 individual sequences, which would have been too time-consuming for Bayesian analysis. For some species, different individuals were included for different sequence datasets, owing to the absence of the previously selected individual in a particular dataset. The newly chosen individual was chosen from the same specific group as the individual it replaced. The GTR+I+Γ model was used in MrBayes, with six substitution types (“nst = 6”) and rate variation across sites modelled using a gamma distribution, with a proportion of sites being invariant (rates = “invgamma”). The Markov chain Monte Carlo search was run with 4 chains for 20 million generations, with trees being sampled every 100 generations. We assessed the convergence of runs and chose to discard the first 2 million generations using Tracer [33].

### Estimation of molecular divergence times

The estimation of divergence times was conducted with BEAST v1.7.2 [34] using all taxa available for the four investigated loci (nITS, nETS, *phantastica*, and concatenated cpDNA) and thus, excluding *F. hupehensis*. The two oldest fossils that could be reliably attributed to the genus were found in the southeast of North America (Middle Eocene - 39–49 mya, [35], [36], [37]), and were used as a prior for the root node age. Normal prior age distribution was assigned to the root node for the genus, which resulted in a mean age calibration of 44 mya (SD = 3.5 mya); for uncertainty, we considered the entirety of the 95% confidence interval (38.2 to 49.8 mya). In addition, a second fossil, namely pollen representative of the morphology of the extant taxon *Fraxinus angustifolia* from the Upper Miocene in Europe (12 mya) [38], was used to set the minimum age (uniform distribution) for the rise of European ashes. The GTR+I+Γ model was used, implicating six substitution types with rate variation across sites modelled using a gamma distribution, and the proportion of sites that were invariant was used with base frequencies estimated for all partitions [34]. A random local clock model was further implemented with a Yule process as the tree prior. Markov chain Monte Carlo searches were run for 150 million generations. We assessed the convergence of runs and chose to discard the first 15 million generations using Tracer [33]. Moreover, for more precise inference of the divergence times for the different loci, a prior was set on the ITS substitution rate. In accordance with Kay et al. [39], we selected a truncated normal distribution with a mean of 2.15×10^−9^ substitutions per site per year and a standard deviation of 3.2×10^−9^, which is a conservative approach given the relative size of this latter value. These searches achieved sufficient mixing as assessed by the high effective sample size values for most parameters, reaching plateaus for divergence time estimates over generations after burn-in, and repeatability of results over multiple independent runs. The results were combined manually, visualized with FigTree v1.3.1 [40], and the section Dipetalae was set as an outgroup. The rates of diversification were then evaluated using default parameters in the Lineage Through Time (LTT) plot from Tracer v1.5 [33], and subsequent statistics were calculated with R [41] using the package APE [42].

### Biogeographic history

The dispersal-extinction-cladogenesis (DEC) model implemented in Lagrange v.20120508 [43] was used to infer the biogeographic history. A geographical range matrix coding each species as present or absent in each of four geographical areas (North America, Asia, Europe, North Africa) was constructed. Species that inhabit more than one geographical area were coded accordingly. Thus, the Lagrange analysis was based on this geographical matrix, and the divergence times calibrated from the BIM consensus tree were obtained from molecular dating with branch lengths equal to mean ages and a root age of 45.4 mya. The dispersal connections between areas were parameterized on the basis of documented land-bridge connections (www.scotese.com) and cooling periods of the Cenozoic [44]. The detailed matrices are shown in Figure S1.

### Reconstruction of floral evolution

Floral evolution was traced onto the phylogenetic tree that resulted from the BIM and BEAST analyses of the combined dataset (cpDNA, ETS, ITS, and *phantastica*) using Mesquite [45]. Parsimony and ML reconstructions were conducted using default parameters in Mesquite [45], with one of the three following states scored for each of the extant species: complete flower (calyx+corolla), incomplete flower (loss of corolla or calyx), or naked flower (no calyx or corolla). Given the lesser ambiguity in the reconstruction of the evolution of floral morphology than that obtained with MP, only the ML reconstruction was mapped onto the phylogenetic tree. Ancestral states of floral characters were estimated using a stochastic model of evolution [46], setting the model to Mk1 (“Markov k-state 1 parameter model”) in Mesquite [45].

## Results

The levels of sequence polymorphism and tree resolution are shown in Table 2 for each genomic region analysed. Given that ML trees and BIM trees were largely congruent, only the Bayesian trees are presented, with the ML bootstrap values added onto the branch lengths. ML trees that include all the individuals for ETS, ITS and *phantastica* datasets are shown in Figures S2, S3 and S4, respectively.

**Table 2:** Sequence variation and parameters of phylogenetic analyses for the various datasets used to estimate the phylogeny of *Fraxinus*.

Sequence dataset	Number of sequences(*Fraxinus*)	Length	Number of variable sites (%)	Number of informative sites (%)	Number of nodes with support	
					PP≥0.50	PP≥0.90
cpDNA	276	1721	223 (12.9)	94 (5.5)	19	6
nITS	118	676	170 (25.1)	142 (21.0)	34	27
nETS	260	480	170(35.4)	112 (23.3)	26	16
*phantastica*	225	627	146 (23.3)	48 (7.7)	17	15
Combined	-	3504	709 (20.2)	396 (11.3)	36	32

PP≥0.50, number of nodes with posterior probability ≥0.50 in Bayesian analyses; PP≥0.90, number of nodes with posterior probability ≥0.90 in Bayesian analyses. Only internal nodes to the genus *Fraxinus* were considered for the estimation of statistical support.

### Analysis of separate datasets

The trees for each of the loci are shown in Figures S5, S6, S7 and S8. Briefly, the sections were well resolved by cpDNA data, but the BIM tree showed moderate support for the branches leading to taxonomical sections (Fig. S6). On average, the phylogenies obtained with the cpDNA intergenic spacer sequences were not as well supported as those using nuclear DNA sequences. This is likely related to the generally lower sequence divergence observed for the cpDNA loci (Table 2). In the BIM nETS tree (Fig. S7), the sections were well resolved and it showed strong support for the branches underlying the different sections of the genus, like for the BIM nITS tree (Fig. S8). This tree was generally congruent with a previous study [4], except for the placement of *F. platypoda* and the section *Pauciflorae*. Finally, the BIM tree for the *phantastica* dataset (Fig. S9) showed high support for the monophyly of all recognized sections (all PP = 1.0), except for the section *Ornus*, in which *F. micrantha* and *F. griffithii* were not included.

### Analysis of the combined dataset (cpDNA, nITS, nETS and phantastica)

When considering only the strongly (PP≥0.90) and moderately (PP≥0.70) supported nodes, the cpDNA tree showed little conflict with the trees produced by the analyses of nuclear DNA loci. Only a few minor incongruences were observed in the placement of some species. For example, *F. floribunda* and *F. griffithii* formed a sister clade separate from all other species of the genus. These incongruences may have been due to incomplete lineage sorting, homoplasy, or very low levels of sequence divergence among the cpDNA sequences of Oleaceae. These minor misplacements may also represent artefacts derived from possible misidentification of specimens in arboreta and herbaria.

**Figure 1:**
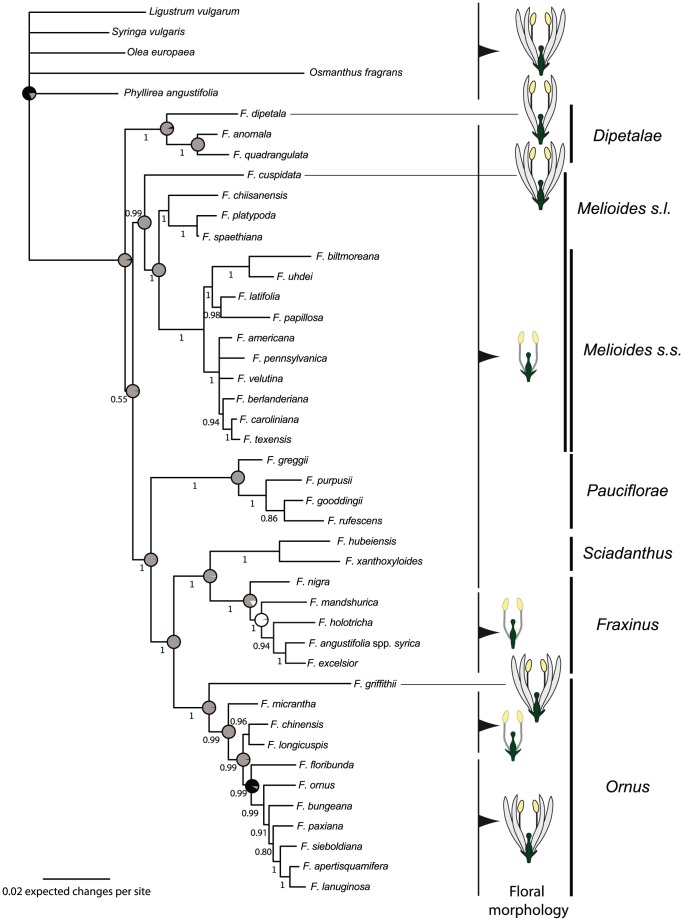
Phylogenetic tree resulting from BIM analysis of the combined dataset (cpDNA, ETS, ITS and *phantastica*). Posterior probabilities ≥0.50 are indicated below the branches. Tree nodes that involve floral changes and the relative likelihoods of floral states are indicated by circles: black, complete flower (calyx+corolla); grey, incomplete flower (loss of corolla or calyx); white, naked flower (no calyx or corolla). On the right, schematic floral character states are shown at the tip of the branches. On the right, sections are indicated by vertical bars; the dotted line indicates the occurrence of *F. platypoda*, previously placed in the section *Fraxinus* [4], but here shown to be closely related to the *incertae sedis* species.

The genus appeared to be divided into three main groups (Fig. 1). The section Dipetalae was monophyletic (PP = 1.0) and a sister group to all other sections of the genus, but this relationship had much lower support (PP = 0.55). All other sections were monophyletic (PP = 1.0), and the section *Fraxinus* was closely associated with the section *Sciadanthus* (PP = 1.0); they formed a sister group to the section *Ornus* (all PP = 1.0). The group formed by these sections had the section *Pauciflorae* as a sister group (PP = 1.0). The other part of the tree grouped together the species of the section *Melioides sensu lato*. In this group, *F. cuspidata* was in a basal position (PP = 1.0). The section *Melioides sensu stricto* was divided into two groups (PP = 1.0 for both groups). One of these groups included a polytomy (*F. americana*, *F. pennsylvanica*, and *F. velutina*) and a group that comprised *F. berlanderiana*, *F. caroliniana*, and *F. texensis* (PP = 0.94). The other group (PP = 1.0) included *F. biltmoreana* and *F. uhdei* in a subgroup (PP = 1.0), with *F. latifolia* and *F. papillosa* (PP = 0.98) being found in the other subgroup.

### Molecular dating

**Figure 2:**
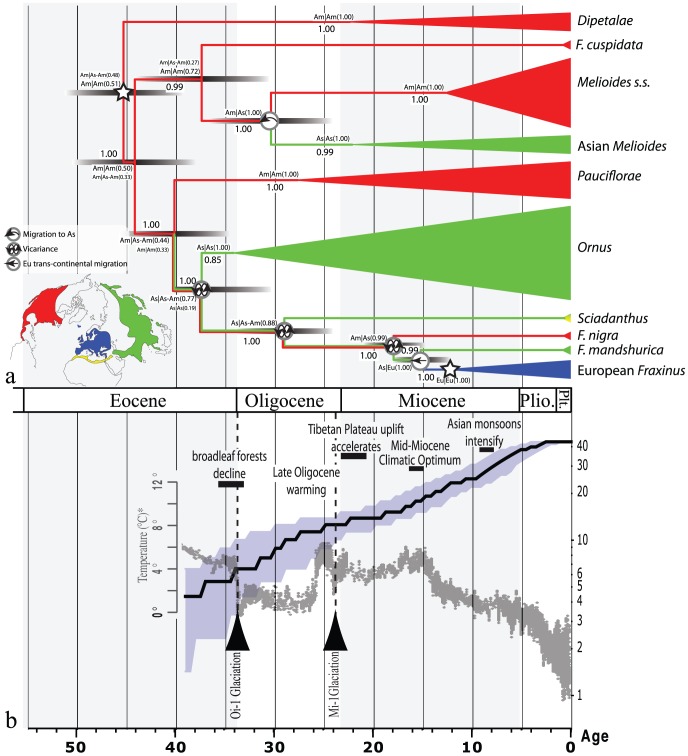
a. Chronogram generated from BEAST analysis and estimations of times of divergence and diversification of sections in *Fraxinus* (in mya), and showing the results from Lagrange analysis. Bars at nodes represent the 95% confidence interval; stars indicate the calibration nodes. Map showing the colours used to identify the areas of occurrence of *Fraxinus* species: red, North America (Am); green, Asia (As); blue, Europe (Eu); yellow, North Africa. The same colours were used on the tree to indicate the reconstructed geographic range across the branches and nodes. For the Lagrange results, a slash indicates the split of areas into two daughter lineages, namely, left/right, where “up” and “down” are the ranges inherited by each descendant branch. The values in brackets represent relative probabilities. When a node has alternative scenarios within 2 log-likelihood units of the optimal reconstruction, the relative probability (fraction of the global likelihood) for the optimal reconstruction is indicated with a smaller font. Circles on nodes indicate migration events or vicariance processes as inferred according to the Lagrange analysis. b. Semilogarithmic Lineage Through Time (LTT) plot (solid line) averaged over 135,000 posterior trees from the BIM analysis: right axis, the solid line represents the cumulative number of lineages and the blue area, the 95% confidence interval; in dark grey, plot of the global deep-sea temperature and its variance, with temperature estimates, geological and biological events reported elsewhere [44]; age in millions of years.

The divergence and diversification times estimated for each section and major groups of the genus are shown in Table 3 and Figure 2a. A minimum age of 45.4±3.5 mya was obtained for the divergence of the section Dipetalae. The divergence times for four of the six major groups (sections *Dipetalae*, *Melioides sensu lato*, *Pauciflorae* and *Ornus*) date back to the middle and late Eocene (35–52 mya), whereas divergence was considered to have occurred during the Oligocene and Miocene for two groups (sections *Sciadanthus* and *Fraxinus*). The split between the American and Asian lineages of the section *Melioides sensus lato* was assumed to have also occurred during the Oligocene. The times for diversification (i.e., the date of the node for the last common ancestor) of four of the seven taxonomical groups (sections *Dipetalae*, *Melioides s.s.*, *Fraxinus*, and the Asian *Melioides* species) were shown to have been during the Miocene (5.3–23 mya) (Figure 2a). The sections *Ornus* and *Pauciflorae* diversified during the upper and lower Oligocene, respectively. All of the inter-continental migrations that led to divergence events were estimated to have occurred before the Miocene, except in the section *Fraxinus*, for which the divergence that led to the North American *F. nigra* from its Eurasian ancestor was postulated to have occurred during the Miocene (Fig. 2a). In general, no clear relationship was found between global temperature changes and the diversification rate over such a large period of time: the cumulative number of divergence events showed a more or less linear increase over time, whereas several large temperature shifts were observed during the past 40 mya (Fig. 2b). The LTT plot showed a decrease in the diversification rates between ≈17 and 25 mya (Fig. 2b), which was not significantly supported by the MCCR test [47] (*P* = 0.153), but was significant using the γ statistic of Pybus and Harvey [47] (*P* = 0.049). According to the test for rate variation using the delta-AICrc statistic [48], the most probable model to fit the diversification rates was the yule2rates, with a shift in rate at ≈5 mya. This model assumes that the group has diversified under speciation rate r1 until time st, at which point the speciation rate shifts to a new rate r2. Thus, overall, the diversification rate was quite constant through time, except more recently during the Pleistocene.

**Table 3:** Estimated times for observed divergence and diversification events in the genus *Fraxinus*.

Node description^1^	Time period	Age ± S.D. (mya)
Divergence of the section *Dipetalae*	Eocene	45.4±1.8
Diversification of the section *Dipetalae*	Oligocene	24.4±1.8
Divergence of the section *Melioides sensu lato*	Eocene	44.2±1.8
Divergence of the section *Melioides sensu stricto*	Eocene	30.4±1.8
Diversification of the section *Melioides s.s.*	Miocene	12.9±1.5
Diversification of the Asian species of the section *Melioides s.l.*	Oligocene	23.7±1.8
Divergence of the section *Ornus*	Eocene	38.5±1.7
Diversification of the section *Ornus*	Oligocene	34.1±1.7
Divergence of the section *Pauciflorae*	Eocene	40.2±1.7
Diversification of the section *Pauciflorae*	Oligocene	28.9±1.9
Divergence of the section *Sciadanthus*	Miocene	28.7±1.6
Divergence of *F. nigra*	Oligocene	18.0±1.3
Divergence of *F. mandshurica*	Miocene	15.3±1.1
Diversification of European *Fraxinus*	Miocene	12.9±0.8

1 With reference to Fig. 1.

### Biogeographical reconstruction

**Figure 3:**
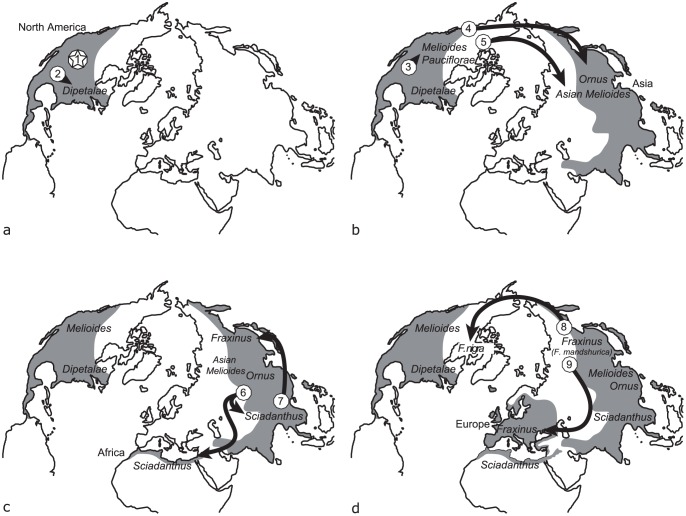
Map illustrating major events in the biogeographic history of the genus *Fraxinus*. (a) The genus likely appeared in North America (1), the section *Dipetalae* diverging early (2). (b) Diversifications leading to the sections *Melioides* and later *Pauciflorae* (3) in North America were followed by migrations to Asia, leading to the sections *Ornus* (4) and to the Asian species of the section *Melioides* (5). (c) The section *Sciadanthus* expanded in Eurasia and Africa (6), (d) followed by the divergence of the Asian section *Fraxinus* (7) and the migration of a lineage leading to *F. nigra* in North America (8). More recently, the differentiation of *F. mandshurica* in Asia and transcontinental expansion of the lineage towards Europe (9) resulted in the observed geographical diversity of the section *Fraxinus* throughout Eurasia.

The results of the Lagrange analyses mapped on the dated phylogeny and a graphical representation of major historical events are shown in Fig. 2a and Fig. 3, respectively. The North American origin of the genus was confirmed (relative probability = 0.51), despite the fact that an ancient vicariance effect between North America and Asia was also retrieved with marginally lower probability (relative probability = 0.48). The analysis inferred colonization events in the section *Melioides* (relative probability = 1.0), resulting in lineages leading to the species *F. spaethiana*, *F. platypoda*, and *F. chiisanensis*. The geographical history of the other Eurasian sections was more complex, with two divergence events in Asia resulting from vicariance, one leading to the section *Ornus* (relative probability = 0.77) and the other to the section *Sciadanthus*, represented only by *F. xanthoxyloides* in our analysis (relative probability = 0.88). Two additional divergence events were inferred in the section *Fraxinus*. Whereas one was suggestive of allopatric divergence that resulted in the lineage leading to *F. nigra* (relative probability = 0.99), the other event implicating the colonization of Europe resulted in the differentiation of three species (*F. excelsior*, *F. angustifolia* and *F. holotricha*) (relative probability = 1.0).

### Reconstruction of evolution of floral morphology

Due to the congruence of results using either the BIM or the BEAST tree, the reconstruction of the evolution of floral morphology is reported in details from the BIM analysis (Fig. 1) while the BEAST tree is reported in Figure S9. The BIM reconstruction that showed the highest likelihood (−log L = 24.68) indicated that there was an ancient state of incomplete flowers at the time of the common ancestor of the genus, with one loss of floral pieces in the section *Fraxinus*, and four reversions from incomplete flowers to complete flowers. There was uncertainty relative to whether a reversion in *F. nigra* or a loss of calyx in Eurasian species occurred, depending on whether a naked or incomplete flower was present at the root of the sections *Sciadanthus* and *Fraxinus*. However, despite the relatively low number of modifications, the floral morphology was not uniform within taxonomical sections. For example, even though the section *Ornus* mostly comprises species with complete flowers, *F. chinensis*, *F. micrantha*, and *F. longicuspis* harbour flowers without a corolla (Fig. 1). In the same way, *F. cuspidata* displays complete flowers in the section *Melioides sensu lato*, which is a group otherwise characterised by flowers without a corolla; likewise, *F. dipetala* displays flowers with only two petals, which is a unique feature in the genus (Fig. 1). Moreover, the extensive sharing of floral morphology among different sections of the genus without any particular correlation with the taxonomic relationships (e.g. incomplete flowers, lacking a corolla but with a calyx, were observed in almost all species of sections *Dipetalae*, *Melioides sensu lato*, *Pauciflorae* and *Sciadanthus*) clearly indicates that floral morphology has not evolved along a single, linear trajectory and that its evolutionary history shows little congruence with the phylogeny of the genus.

## Discussion

### The phylogeny of the genus *Fraxinus*

The phylogenetic tree that resulted from the analysis of the combined dataset had higher resolution than the trees based on analyses of separate datasets and the previous nITS trees of Wallander [4] and Jeandroz et al. [8]. It resulted in a notable improvement in delineation of the relationships among the different sections of the genus. Despite the same placement as in Wallander [4] and Jeandroz et al. [8], the support for the position of the section *Dipetalae* at the root of the tree was only moderate. The section *Fraxinus*, which previously formed a polytomy with the sections *Sciadanthus* and *Melioides* [8], was closely related to the section *Sciadanthus* and, to a lesser extent, to the section *Ornus*. While the section *Pauciflorae* was not considered by Jeandroz et al. [8], our results agree with the previous placement of this section as the sister group to the Old World sections [4].

In all analyses, *F. platypoda* appeared to be closely related to *F. spaethiana*, which was previously considered as an *incertae sedis* species [4]. This close relationship was initially suggested by Nakaike [49] and Wei and Green [50] on the basis of morphological characters. Moreover, *F. spaethiana* has been recognized by some researchers as a synonym of *F. platypoda*, which is in accordance with our results [51]. The close relatedness of *F. chiisanensis* to this species pair, with moderate to high support for all datasets, suggested that these three Asian species should be grouped into the same section. Given their well-supported relationship with taxa from the section *Melioides*, we suggest that these three species (*F. chiisanensis*, *F. platypoda*, and *F. spaethiana*) and *F. cuspidata* should be included in an expanded section *Melioides*, in agreement with the historical placement of *F. platypoda* in this section (prior to Wallander [4]; [50]). The close relationship observed between these species and the section *Melioides* was not in conflict with the results of Wallander [4], where a large polytomy was obtained at this level.

The section *Melioides* comprises 10 recognized species, although more than 28 have been described, [4]. It is the second largest section in terms of the number of species, the largest one being *Ornus* with 15 species. The section *Melioides* was poorly resolved in the analysis based on the combined dataset, as shown by the polytomy of *F. americana*, *F. velutina*, and *F. pennsylvanica*. Given that this polytomy included different species depending on which dataset was considered, we suggest that these species experienced rapid radiation, or that they have exchanged genes recently. Previous molecular phylogenies had outlined a polytomy involving *F. pennsylvanica*, *F. velutina*, and *F. tomentosa* [8], or *F. caroliniana*, *F. pennsylvanica*, *F. texensis*, *F. velutina*, *F. profunda*, and *F. berlanderiana* [4]. The observed low differentiation at the genetic level parallels similar trends at the morphological level, which resulted in either the treatment of several species as synonyms [16], [52] or in the delineation of morphological taxa of various nature (see [53], [54], [55], [56], [57], [58], [59], [60]). In the absence of morphological or molecular evidence regarding the specific status of American taxa from the section *Melioides* and the status of their reproductive isolation [4], it remains difficult to estimate precisely how much genetic divergence exists among these species.

Further studies that involve artificial crosses, as well as morphological and molecular characterization of trees sampled in potential zones of contact, should help clarify whether some of these species should be treated as subspecific taxa and if they are actively hybridizing. If either of these criteria applies, the correct estimation of phylogenetic relationships among species would represent a significant challenge, even if additional sequence data were obtained.

### The evolution of floral morphology

When tracing the evolution of floral characters onto the present phylogeny, we observed that floral morphology varied even within taxonomic groups. As underlined by Regal [61], and as previously suggested for *Fraxinus* [4], floral morphology has likely been affected by ecological factors, such as the pollination system. The loss of corolla has been associated with a transition to an anemophilous pollination system, and sometimes to dioecy [4]. However, on the basis of the present phylogeny, we did not observe any systematic relationship between floral morphology and the habitat preferences of species (desert or temperate regions with predominant wind pollination *vs.* subtropical areas and higher diversity of pollinators); this result suggests that the type of pollination could be an auxiliary factor in the evolution of *Fraxinus*. Elucidation of the evolutionary relevance of these factors will require more ecological and biological studies of diverse species within the genus.

### The biogeographic history of the genus *Fraxinus* and implications for other tree genera

Our results agree with the hypothesis of an Eocene (between 34 to 56 mya) North American origin for the genus [8], [62], [63] (Fig. 3a), as indicated by the more basal position of the species or groups of species found in North America (sections *Dipetalae*, *Melioides*, and *Pauciflorae*). The view of a North American origin of the genus is reinforced by evidence from the fossil record, given that the two oldest fossils that can be reliably attributed to the genus were found in the southeast of North America [35], [36], [37].

The present estimates from molecular dating are also congruent with those based on cpDNA sequences for the diversification of the family Oleaceae [63]. Fossils for which the specific status is unclear, including a putative leaflet fossil of *F. juglandina* from the Eocene era in China, which would be morphologically close to *F. ornus* [64], and samaras of *F. oishii* from the Miocene era in Korea, which would be morphologically close to *F. mandshurica* [65], could dramatically increase the inferred age of the genus if used as landmarks, and put the estimate well at odds with the conventional history of the family Oleaceae [62], [63]. These fossils might also be representative of morphological features of extinct lineages that evolved repeatedly in the genus. Owing to these uncertainties, they were not considered as landmarks in the estimation of divergence times.

The estimated diversification rates were quite consistent over time, despite large shifts in global temperature during the past 40 mya (Fig. 2b). It is possible that the diversification rate was underestimated during past periods of warmer climate, for instance, around the Mid-Miocene Climatic Optimum, given that extinct taxa could not be accounted for and that this bias should be more severe as one moves back in time. However, the number of divergence events leading to extant taxa appeared to vary extensively in each section. These sections were well supported by the phylogeny, and with the exception of the cosmopolitan section *Fraxinus* (which is found in Europe, East Asia, and the east of North America), all other sections of the genus are found mostly in only one of these great areas of endemism [66]. Thus, a number of factors more specific to the geographical locations of these sections have likely affected their evolution. Palaeobiogeographical inferences at the regional level are presented below; they are likely applicable to other temperate tree genera that exhibit similar geographic distributions.

A larger number of species and higher diversification rates were observed for the sections *Ornus* (15 species, 0.51 sp./mya) and *Melioides sensu lato* (10 species, 0.32 sp./mya) than in the sister sections *Fraxinus* and *Sciadanthus* (six species, 0.23 sp./mya and two species, 0.12 sp./mya, respectively). This trend is in agreement with the view that the regions where the sections *Ornus* and *Melioides sensu lato* are found, namely, East Asia and North America, respectively, were less influenced by the drying climate and glaciations that affected Europe and the west of North America, respectively, which may have led to the survival of marginally more plant species [66], including a larger number of species surviving in East Asia (for a review, see [67]).

However, other factors that have likely affected the number of species in a particular section should be considered, including the occurrence of vicariance-inducing factors, such as topographical and climatic variation that led to the fragmentation of natural distributions. For instance, it seems reasonable to assume that major geological events such as the Tibetan Plateau uplift of the early Miocene created new ecological niches that the Asian species could have invaded, or that the Mid-Miocene Climatic Optimum represented an opportunity for Asian species to colonize the newly contiguous European landmass to the west, which resulted in divergence events and new lineages. It should also be noted that, since the Miocene, major events that one would expect to have affected species in particular sections of the genus (depending of the location of these events) had no noticeable influence on the general diversification rate (see Fig. 2b).

Given that the most ancient fossils of *Fraxinus* representative of the morphology of European extant taxa (including pollen) date back to the Upper Miocene (11.6–5.3 mya) [68], and considering the history depicted by the phylogeny obtained herein, it is clear that the climate and conformation of inter-continental land bridges have varied extensively since then, with variations in mean annual temperature of up to 6°C [44] and variations in sea level of up to 60 meters [69]. These changing conditions likely provided opportunities for extensive shifts in natural distributions, including more or less ancient inter-continental spread. It is generally accepted that two dispersal pathways existed between North America and Eurasia during the Tertiary: the Atlantic and Pacific tracks, respectively through a North Atlantic bridge and through Beringia [67], [70]. In agreement with previous reports [8], our results suggest that the Pacific track was involved in the early emergence of both the lineage leading to *F. chiisanensis*, *F. platypoda*, and *F. spaethiana* and the section *Ornus* from a North American ancestor (Fig. 3b), because of the absence of European species in the section *Melioides sensu lato* and given the geographic proximity. Regarding the inferred allopatric divergence that led to the emergence of the North American *F. nigra* at the end of the upper Miocene (Fig. 3d), in the section *Fraxinus* that is otherwise composed of species from Eurasia, it is difficult to determinate whether the migration proceeded from Asia or from Europe. Because *F. nigra* is currently found only in the east of North America, one might argue that its ancestor is from Europe. However, given the palaeogeographical reconstruction described herein, the allopatric speciation leading to the *F. nigra* lineage likely proceeded from Asia through the Pacific track. Given their high levels of morphological and ecological similarity, *F. mandshurica* and *F. nigra* are also sometimes referred to as “geographical races” [71]. According to this more credible scenario, the isolation of the lineage leading to *F. nigra* in North America could be related to the contraction of the Northern Hemisphere boreotropical forest at the end of the Oligocene [66], [67]. Indeed, such North American - Asian disjunct taxa (e.g. *Liriodendron*, *Liquidambar*, and *Magnolia*, review in [72]) are thought to be relicts of the temperate forests of the Northern Hemisphere, which reached their maximum development during the warm period of the Tertiary (Axelrod, 1975 in [72]) and would have declined during the last 30 mya [66]. Such diversification patterns that suggest inter-continental allopatric speciation appear to be common in temperate forest tree genera, given that the same genera are often found in several areas of endemism around the world [66].

Whereas *F. nigra* is the only North American species in the section *Fraxinus*, all of the other three species of the section are found in Eurasia and are arranged in a distribution pattern typical of geographic speciation induced by the effects of colonization or fragmentation inducing vicariance (Fig. 3d). As a sister taxon to this Eurasian group, *F. mandshurica* has a large distribution in East Asia, which covers 23 degrees of latitude and 46 degrees of longitude in China and the Russian Far East [71]. It is followed westward by *F. holotricha*, with an endemic distribution in the Balkan Peninsula. The species *F. excelsior* is found further west throughout Europe, except in the Mediterranean region, to which *F. angustifolia* is mostly restricted. These putative speciations associated with specific geographic areas are consistent with previous reports [8] and would have been driven by climatic and geological changes [67], [70], such as the contraction of the boreotropical forest found in the Northern Hemisphere during the Tertiary [73]. For example, the Mediterranean species *Quercus suber* would have been affected by Tertiary geological and climatic events in Eurasia that would have lead to the early divergence of intraspecific lineages [74].

These relatively recent speciation events are likely to have been accompanied by reticulate evolution during the early divergence of these ash taxa. Such trend is still observable today where natural hybridization is occurring in zones of contact between taxa (e.g. [16]), underlining the slow evolution of reproductive isolation in ashes. Such a phenomenon has been demonstrated both in conifers and angiosperm trees in Eurasia. For instance, in the conifer genus *Tsuga*, *T. dumosa* (found in the Himalayas) has been shown to be a hybrid taxon [75], while in angiosperm trees, the central Asian species *Populus tristis* is thought to be a hybrid between *P. nigra* and another unknown Asian species [13]. In the perennial shrubs *Paeonia*, it has been shown that the European populations of the Asian species have been replaced by their European×Asian hybrids, probably due to differential selection promoted by Pleistocene climatic changes [76].

In addition, some species such as *F. excelsior* (common ash) could have expanded their southern range during periods with cooler climates, leading to the present relic populations in locations where the climate is now temperate, like in the Elburz Mountains or the adjacent mountain ranges in Turkey. These trends further suggest the occurrence of generally slow speciation process, in temperate tree genera with similar life history, reproductive and demographic attributes as well as large geographical ranges (e.g. [13], [17]). Moreover, perennial plants such as ashes have long and overlapping generation times with often large effective population sizes, which would retard reproductive isolation and speciation [77]. Some *Fraxinus* species (e.g., *F. excelsior*) have a particularly complicated mating system (trioecy: male, female, and hermaphrodite individuals), which may interfere in this respect. Despite the fact that the analysis of *F. excelsior* revealed almost functional dioecy [5], it is possible that the mating system plays a significant role in speciation, as shown for the transition from nondioecy to dioecy [78].

The colonization of new habitats leading to geographic speciation could further be hypothesized for several pairs of taxa in the genus, given that the species involved are phylogenetically closely related when found in parapatry or in much or less close allopatry: for instance, the pairs *F. anomala* and *F. quadrangulata* in the United States, *F. papillosa* and *F. latifolia* as well as *F. xanthoxyloides* and *F. hupehensis* in Asia, and *F. angustifolia* and *F. excelsior* in Europe. Such a repeated pattern of phylogenetic grouping of geographically close species has been noted in an increasing number of other tree genera [17], [79], which further suggests an important role for geographic speciation in promoting the past diversification of temperate and boreal tree taxa. Thereafter, these divergences may have led to ecological specialization, as each pair contains species that may have adapted to contrasting environments: dry *versus* more Mediterranean climates, or temperate *versus* warmer subtropical climates.

Thus, the repeated observations of geographical patterning at various scales in the phylogeny of the genus *Fraxinus* provide strong support for the importance of vicariance and geographic speciation as a central mechanism for diversification in this genus. Together with the noted slow evolution of reproductive isolation and reported instances of reticulate evolution in the genus, these processes could explain much of the patterns of interspecific variation noted at the genus-wide level in *Fraxinus*. These observations are in line with recent reports for other anemophilous and vastly distributed tree genera in the conifers [17] and angiosperms [80], which highlight the central role of vicariance and geographic speciation in driving the emergence of new lineages in temperate and boreal trees.

## Supporting Information

Figure S1 Left: Matrix used for the Lagrange analysis. Right: Palaeogeographic maps showing the intensity of inferred migrations. Palaeomaps redrawn from Nie et al. [81].
(EPS)

Figure S2 Phylogenetic tree resulting from the maximum likelihood (ML) analysis of the nETS dataset, including all sequences. ML bootstrap values are indicated above the branches. Sections according to a previous report [4] are indicated by vertical bars.
(PDF)

Figure S3 Phylogenetic tree resulting from the maximum likelihood (ML) analysis of the nITS dataset, including all sequences. ML bootstrap values are indicated above the branches. Sections according to a previous reports [4] are indicated by vertical bars.
(PDF)

Figure S4 Phylogenetic tree resulting from the maximum likelihood (ML) analysis of the *phantastica* dataset, including all sequences. ML bootstrap values are indicated above the branches. Sections according to a previous report [4] are indicated by vertical bars.
(PDF)

Figure S5 Phylogenetic tree resulting from BIM analysis of the cpDNA dataset (*trnH-psbA* and *rpl32-trnL*). Posterior probabilities ≥0.50 are indicated above the branches, ML bootstrap values are indicated below the branches. Sections according to a previous report [4] are indicated by vertical bars, and the dotted line indicates the occurrence of *F. platypoda*, previously placed in the section *Fraxinus* [4], but here shown to be closely related to the *incertae sedis* species. An asterisk indicates a species that was not clustered with other species of the same section.
(EPS)

Figure S6 Phylogenetic tree resulting from BIM analysis of the nETS dataset. Posterior probabilities ≥0.50 are indicated above the branches, ML bootstrap values are indicated below the branches. Sections according to a previous report [4] are indicated by vertical bars, and the dotted line indicates the occurrence of *F. platypoda*, previously placed in the section *Fraxinus* [4], but here found to be closely related to the *incertae sedis* species.
(PDF)

Figure S7 Phylogenetic tree resulting from BIM analysis of the nITS dataset. Posterior probabilities ≥0.50 are indicated above the branches, ML bootstrap values are indicated below the branches. Sections according to a previous report [4] are indicated by vertical bars, and the dotted line indicates the occurrence of *F. platypoda*, previously placed in the section *Fraxinus* [4], but here found to be closely related to the *incertae sedis* species.
(PDF)

Figure S8 Phylogenetic tree resulting from BIM analysis of the *phantastica* dataset. Posterior probabilities ≥0.50 are indicated above the branches, ML bootstrap values are indicated below the branches. Sections according to a previous report [4] are indicated by vertical bars, and the dotted line indicates the occurrence of *F. platypoda*, previously placed in the section *Fraxinus* [4], but here found to be closely related to the *incertae sedis* species.
(PDF)

Figure S9 Phylogenetic tree resulting from the BEAST analysis of the four datasets (cpDNA – *trnH*-*psbA* and *rpl32*-*trnL*, nETS, nITS and *phantastica*). Bars at nodes represent the 95% confidence interval; posterior probabilities above the branches. Ages in million years before present.
(PDF)

Table S1 *Fraxinus* samples used in this study, herbarium vouchers and newly published DNA sequences. ID stands for identifier; Sample type: Origin of the sample used in this study; A, Arboretum; W, Collected in the wild; H, Herbarium. Vouchers are deposited at the National Herbarium, Muséum National d'Histoire Naturelle, Paris, France (P00729547 to P00729694), or at the Mexico Herbarium (MEXU1032796 to MEXU991880); Arb.: Arboretum.
(DOC)
