# Supplementary material for: The Phylogeny and Biogeographic History of Ashes (Fraxinus, Oleaceae) Highlight the Roles of Migration and Vicariance in the Diversification of Temperate Trees
Source: PLoS One. 2013 Nov 21;8(11):e80431. doi: 10.1371/journal.pone.0080431 (PMC3837005; doi:10.1371/journal.pone.0080431)
Supplement: Figure S2 — Phylogenetic tree resulting from the maximum likelihood (ML) analysis of the nETS dataset, including all sequences. ML bootstrap values are indicated above the branches. Sections according to a previous report [4] are indicated by vertical bars. (PDF) [file pone.0080431.s002.pdf]

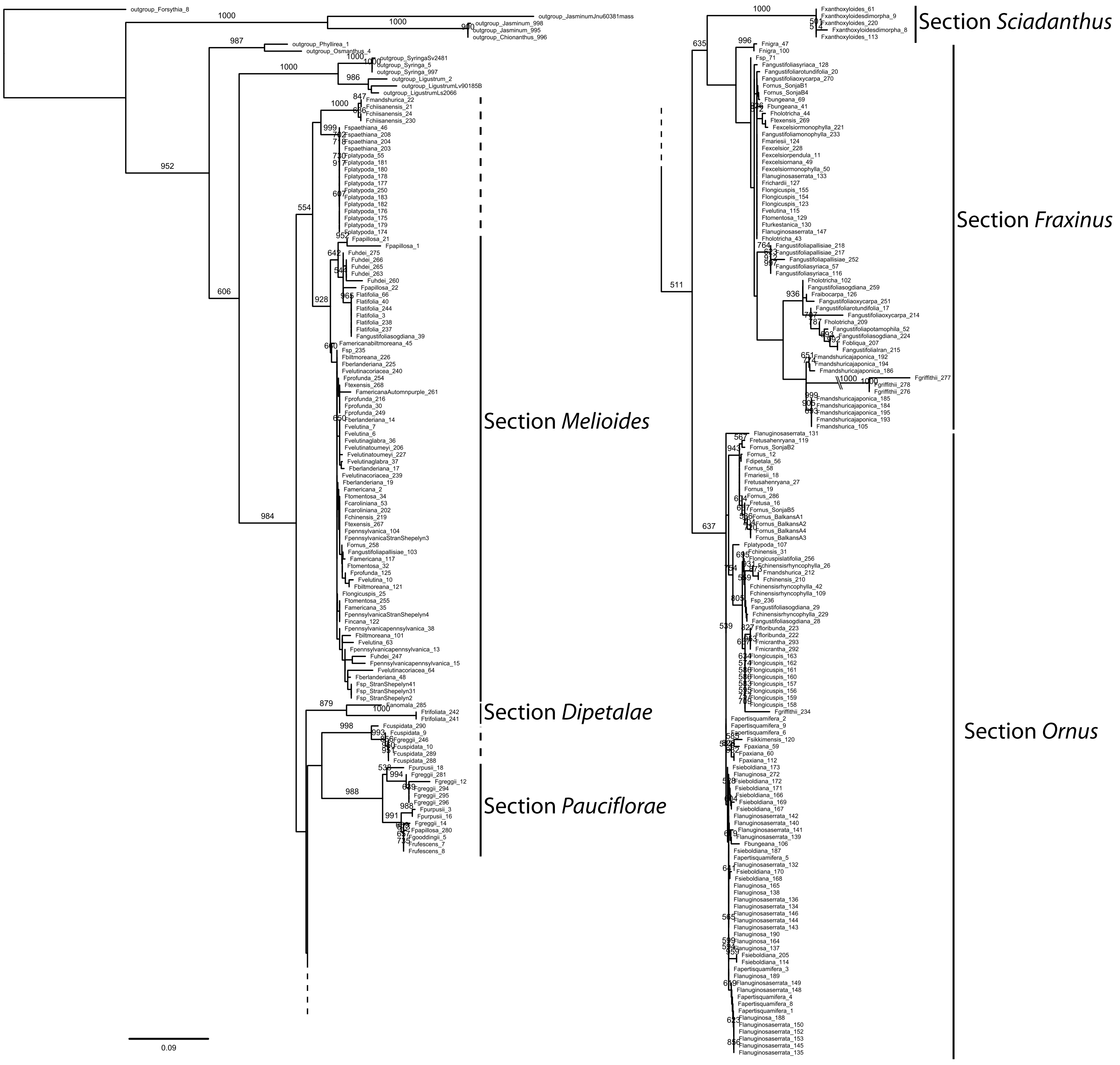
Section *Sciadanthus*
Section *Fraxinus*
Section *Melioides*
Section *Dipetalae*
Section *Ornus*
Section *Pauciflorae*
0.09
