# Supplementary figures and images for: The Phylogeny and Biogeographic History of Ashes (Fraxinus, Oleaceae) Highlight the Roles of Migration and Vicariance in the Diversification of Temperate Trees
Source: PLoS One. 2013 Nov 21;8(11):e80431. doi: 10.1371/journal.pone.0080431 (PMC3837005; doi:10.1371/journal.pone.0080431)

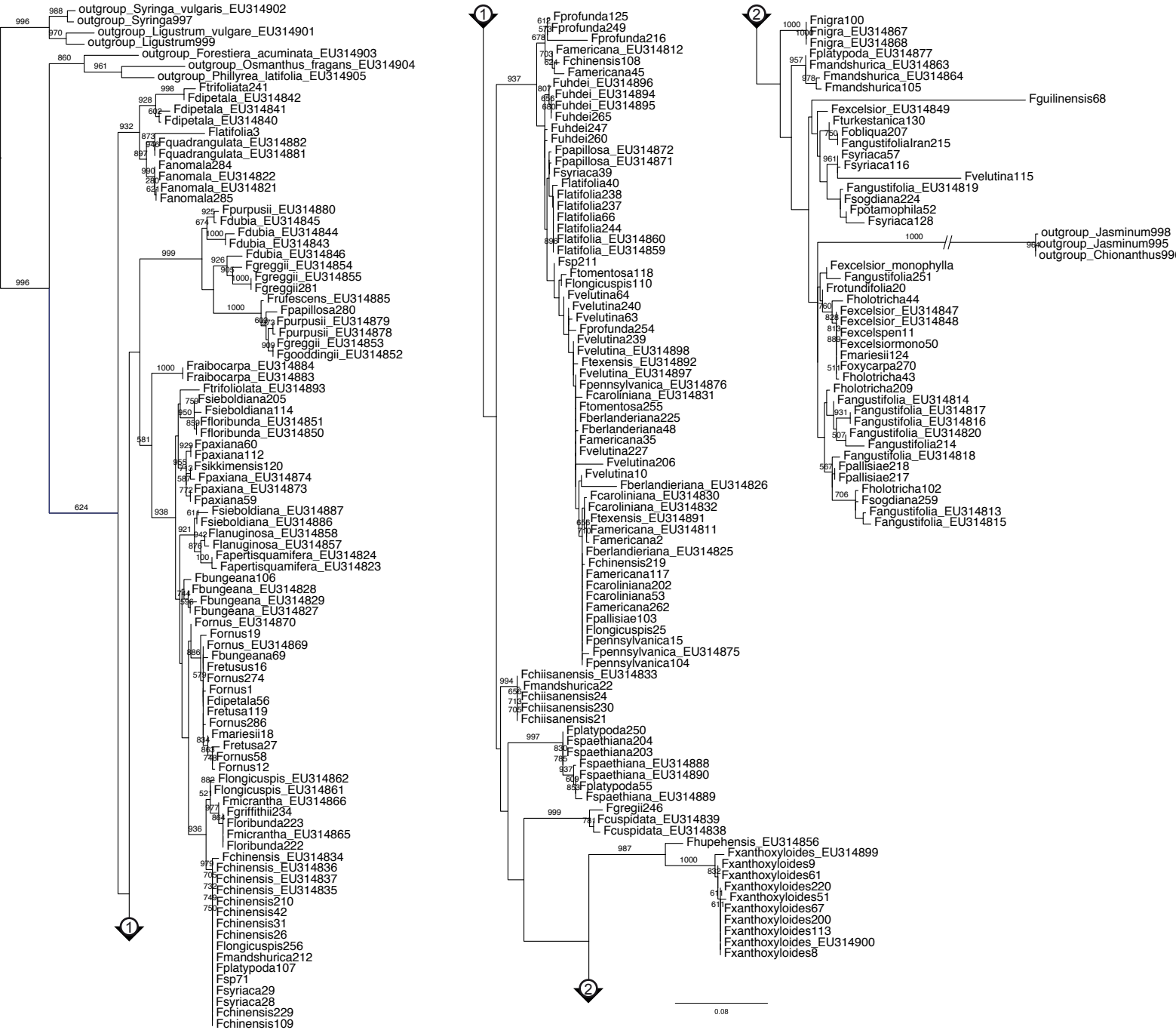

Supplement: Figure S3 — Phylogenetic tree resulting from the maximum likelihood (ML) analysis of the nITS dataset, including all sequences. ML bootstrap values are indicated above the branches. Sections according to a previous reports [4] are indicated by vertical bars. (PDF) [file pone.0080431.s003.pdf]

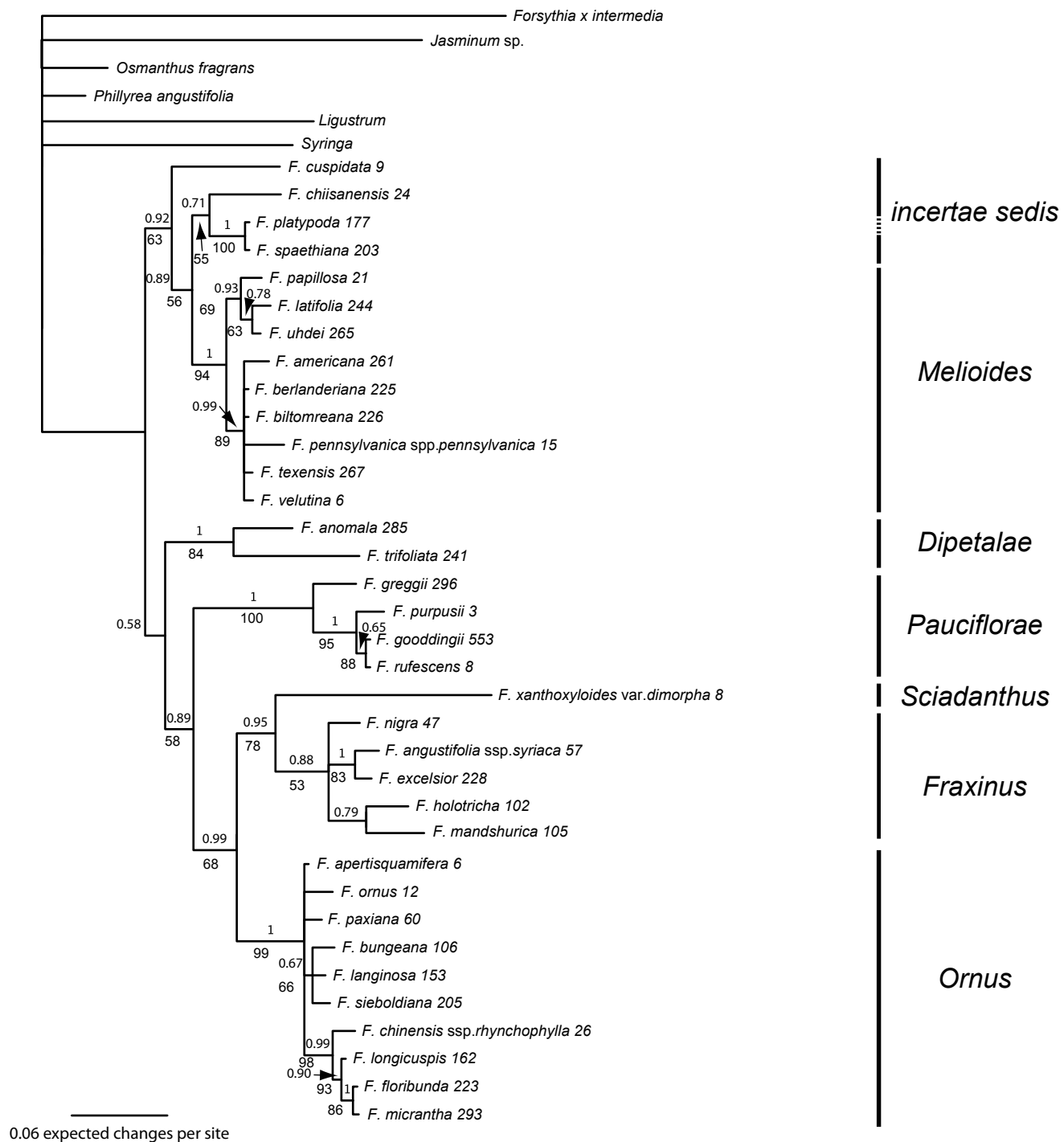

Supplement: Figure S6 — Phylogenetic tree resulting from BIM analysis of the nETS dataset. Posterior probabilities ≥0.50 are indicated above the branches, ML bootstrap values are indicated below the branches. Sections according to a previous report [4] are indicated by vertical bars, and the dotted line indicates the occurrence of F. platypoda, previously placed in the section Fraxinus [4], but here found to be closely related to the incertae sedis species. (PDF) [file pone.0080431.s006.pdf]

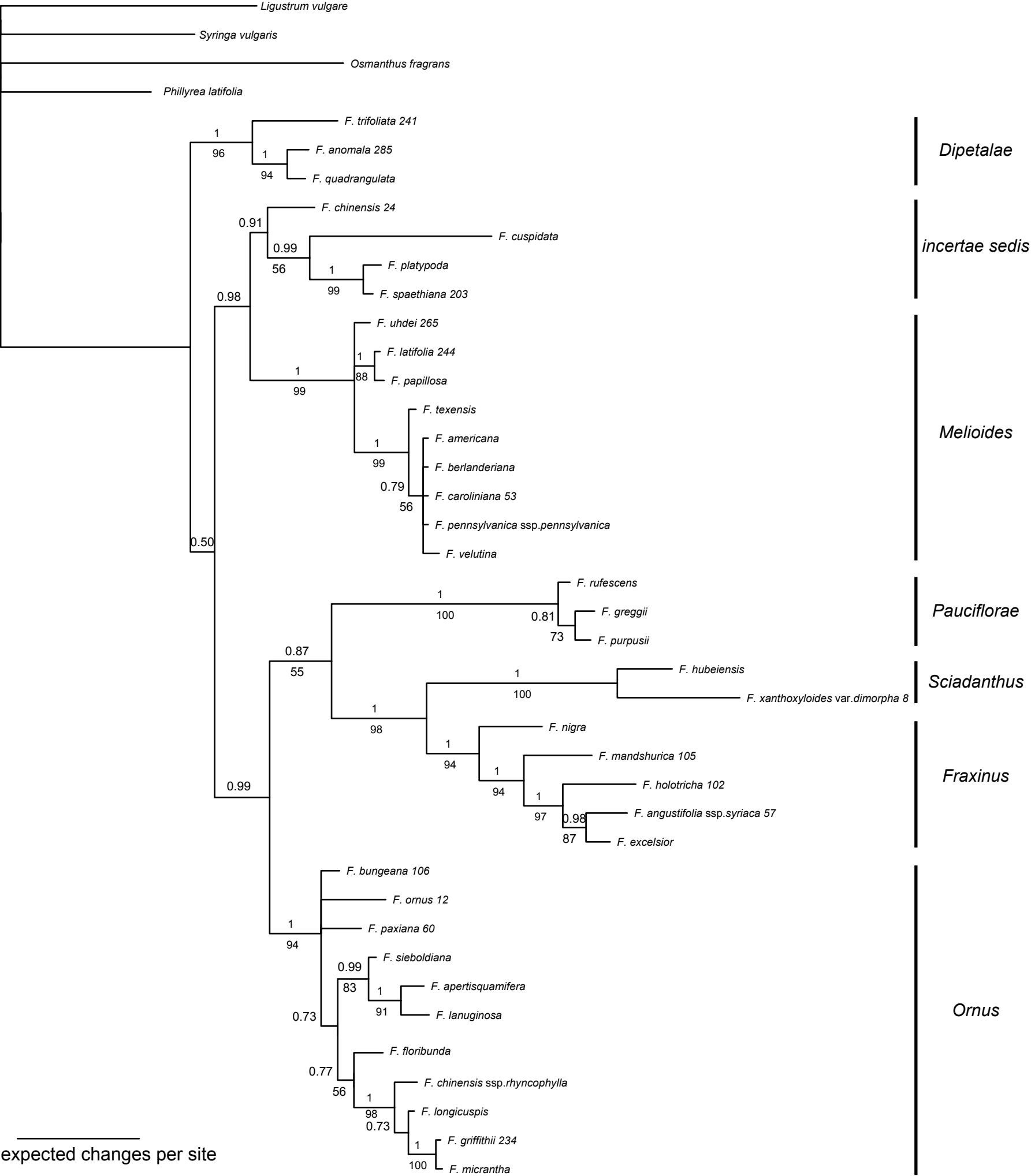

Supplement: Figure S7 — Phylogenetic tree resulting from BIM analysis of the nITS dataset. Posterior probabilities ≥0.50 are indicated above the branches, ML bootstrap values are indicated below the branches. Sections according to a previous report [4] are indicated by vertical bars, and the dotted line indicates the occurrence of F. platypoda, previously placed in the section Fraxinus [4], but here found to be closely related to the incertae sedis species. (PDF) [file pone.0080431.s007.pdf]
