# Supplementary material for: The Phylogeny and Biogeographic History of Ashes (Fraxinus, Oleaceae) Highlight the Roles of Migration and Vicariance in the Diversification of Temperate Trees
Source: PLoS One. 2013 Nov 21;8(11):e80431. doi: 10.1371/journal.pone.0080431 (PMC3837005; doi:10.1371/journal.pone.0080431)
Supplement: Figure S4 — Phylogenetic tree resulting from the maximum likelihood (ML) analysis of the phantastica dataset, including all sequences. ML bootstrap values are indicated above the branches. Sections according to a previous report [4] are indicated by vertical bars. (PDF) [file pone.0080431.s004.pdf]

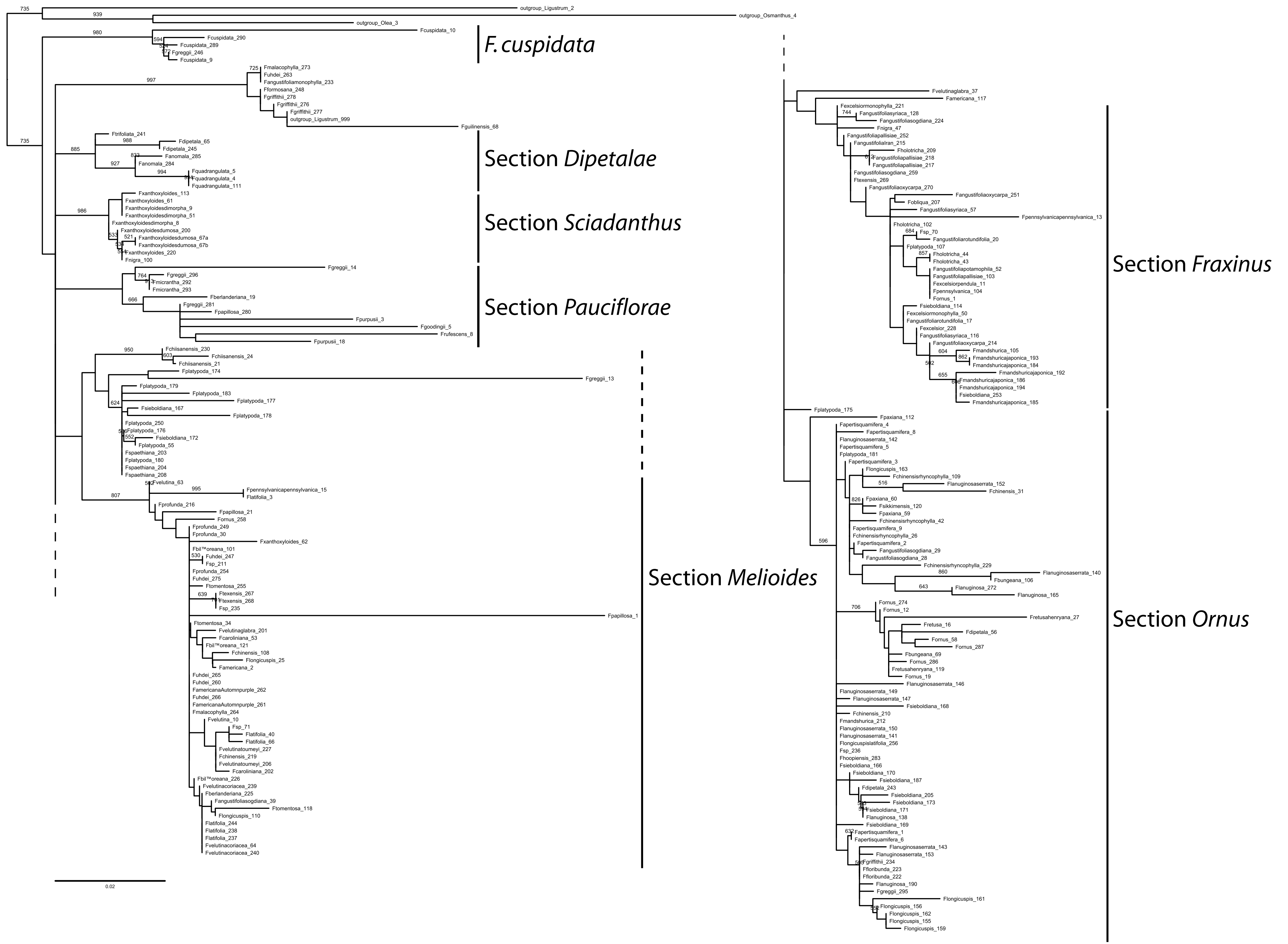
735
939
outgroup_Ligustrum_2
outgroup_Osmanthus_4
outgroup_Olea_3
980
Fcuspidata_10
594
Fcuspidata_290
Fcuspidata_289
Fgreggii_246
Fcuspidata_9
F. cuspidata
997
725
Fmalacophylla_273
Fuhdei_263
Fangustifoliamonophylla_233
Fformosana_248
Fgriffithii_278
Fgriffithii_276
Fgriffithii_277
outgroup_Ligustrum_999
Fguilinensis_68
735
885
Ftrifoliata_241
988
Fdipetala_65
Fdipetala_245
927
Fanomala_285
Fanomala_284
994
Fquadrangulata_5
Fquadrangulata_4
Fquadrangulata_111
Section Dipetalae
986
Fxanthoxyloides_113
Fxanthoxyloides_61
Fxanthoxyloidesdimorpha_9
Fxanthoxyloidesdimorpha_51
Fxanthoxyloidesdimorpha_8
Fxanthoxyloidesdumosa_200
533
521
Fxanthoxyloidesdumosa_67a
Fxanthoxyloidesdumosa_67b
Fxanthoxyloides_220
Fnigra_100
Section Sciadanthus
Fgreggii_14
764
Fgreggii_296
Fmicrantha_292
Fmicrantha_293
666
Fberlanderiana_19
Fgreggii_281
Fpapillosa_280
Fpurpusii_3
Fgoodingii_5
Frufescens_8
Fpurpusii_18
Section Pauciflorae
950
Fchiisanensis_230
603
Fchiisanensis_24
Fchiisanensis_21
Fplatypoda_174
Fgreggii_13
Fplatypoda_179
Fplatypoda_183
624
Fplatypoda_177
Fsieboldiana_167
Fplatypoda_178
Fplatypoda_250
Fplatypoda_176
552
Fsieboldiana_172
Fplatypoda_55
Fspaethiana_203
Fplatypoda_180
Fspaethiana_204
Fspaethiana_208
807
Fvelutina_63
995
Fpennsylvanicapennsylvanica_15
Flatifolia_3
Fprofunda_216
Fpapillosa_21
Fornus_258
Fprofunda_249
Fprofunda_30
Fxanthoxyloides_62
Fbil™oreana_101
530
Fuhdei_247
Fsp_211
Fprofunda_254
Fuhdei_275
Ftomentosa_255
639
Ftexensis_267
Ftexensis_268
Fsp_235
Fpapillosa_1
Ftomentosa_34
Fvelutinaglabra_201
Fcaroliniana_53
Fbil™oreana_121
Fchinensis_108
Flongicuspis_25
Famericana_2
Fuhdei_265
Fuhdei_260
FamericanaAutomnpurple_262
Fuhdei_266
FamericanaAutomnpurple_261
Fmalacophylla_264
Fvelutina_10
Fsp_71
Flatifolia_40
Flatifolia_66
Fvelutinatoumeyi_227
Fchinensis_219
Fvelutinatoumeyi_206
Fcaroliniana_202
Fbil™oreana_226
Fvelutinacoriacea_239
Fberlanderiana_225
Fangustifoliasogdiana_39
Ftomentosa_118
Flongicuspis_110
Flatifolia_244
Flatifolia_238
Flatifolia_237
Fvelutinacoriacea_64
Fvelutinacoriacea_240
Section Melioides
0.02
Fvelutinaglabra_37
Famericana_117
Fexcelsiormonophylla_221
744
Fangustifoliasyriaca_128
Fangustifoliasogdiana_224
Fnigra_47
Fangustifoliapallisiae_252
FangustifoliaIran_215
Fholotricha_209
Fangustifoliapallisiae_218
Fangustifoliapallisiae_217
Fangustifoliasogdiana_259
Ftexensis_269
Fangustifoliaoxycarpa_270
Fangustifoliaoxycarpa_251
Fobliqua_207
Fangustifoliasyriaca_57
Fpennsylvanicapennsylvanica_13
Fholotricha_102
684
Fsp_70
Fangustifoliarotundifolia_20
Fplatypoda_107
857
Fholotricha_44
Fholotricha_43
Fangustifoliapotamophila_52
Fangustifoliapallisiae_103
Fexcelsiorpendula_11
Fpennsylvanica_104
Fornus_1
Fsieboldiana_114
Fexcelsiormonophylla_50
Fangustifoliarotundifolia_17
Fexcelsior_228
Fangustifoliasyriaca_116
Fangustifoliaoxycarpa_214
604
Fmandshurica_105
862
Fmandshuricajaponica_193
Fmandshuricajaponica_184
655
Fmandshuricajaponica_192
Fmandshuricajaponica_186
Fmandshuricajaponica_194
Fsieboldiana_253
Fmandshuricajaponica_185
Section Fraxinus
Fplatypoda_175
Fpaxiana_112
Fapertisquamifera_4
Fapertisquamifera_8
Flanuginosaserrata_142
Fapertisquamifera_5
Fplatypoda_181
Fapertisquamifera_3
Flongicuspis_163
Fchinensisrhyncophylla_109
516
Flanuginosaserrata_152
Fchinensis_31
826
Fpaxiana_60
Fsikkimensis_120
Fpaxiana_59
Fchinensisrhyncophylla_42
Fapertisquamifera_9
Fchinensisrhyncophylla_26
Fapertisquamifera_2
596
Fangustifoliasogdiana_29
Fangustifoliasogdiana_28
Fchinensisrhyncophylla_229
860
Flanuginosaserrata_140
Fbungeana_106
643
Flanuginosa_272
Flanuginosa_165
706
Fornus_274
Fornus_12
Fretusahenryana_27
Fretusa_16
Fdipetala_56
Fornus_58
Fornus_287
Fbungeana_69
Fornus_286
Fretusahenryana_119
Fornus_19
Flanuginosaserrata_146
Flanuginosaserrata_149
Flanuginosaserrata_147
Fsieboldiana_168
Fchinensis_210
Fmandshurica_212
Flanuginosaserrata_150
Flanuginosaserrata_141
Flongicuspislatifolia_256
Fsp_236
Fhoopiensis_283
Fsieboldiana_166
Fsieboldiana_170
Fsieboldiana_187
Fdipetala_243
Fsieboldiana_205
Fsieboldiana_173
Fsieboldiana_171
Flanuginosa_138
Fsieboldiana_169
632
Fapertisquamifera_1
Fapertisquamifera_6
Flanuginosaserrata_143
Flanuginosaserrata_153
Fgriffithii_234
Ffloribunda_223
Ffloribunda_222
Flanuginosa_190
Fgreggii_295
Flongicuspis_161
Flongicuspis_156
Flongicuspis_162
Flongicuspis_155
Flongicuspis_159
Section Ornus
