# Supplementary material for: The Phylogeny and Biogeographic History of Ashes (Fraxinus, Oleaceae) Highlight the Roles of Migration and Vicariance in the Diversification of Temperate Trees
Source: PLoS One. 2013 Nov 21;8(11):e80431. doi: 10.1371/journal.pone.0080431 (PMC3837005; doi:10.1371/journal.pone.0080431)
Supplement: Figure S8 — Phylogenetic tree resulting from BIM analysis of the phantastica dataset. Posterior probabilities ≥0.50 are indicated above the branches, ML bootstrap values are indicated below the branches. Sections according to a previous report [4] are indicated by vertical bars, and the dotted line indicates the occurrence of F. platypoda, previously placed in the section Fraxinus [4], but here found to be closely related to the incertae sedis species. (PDF) [file pone.0080431.s008.pdf]

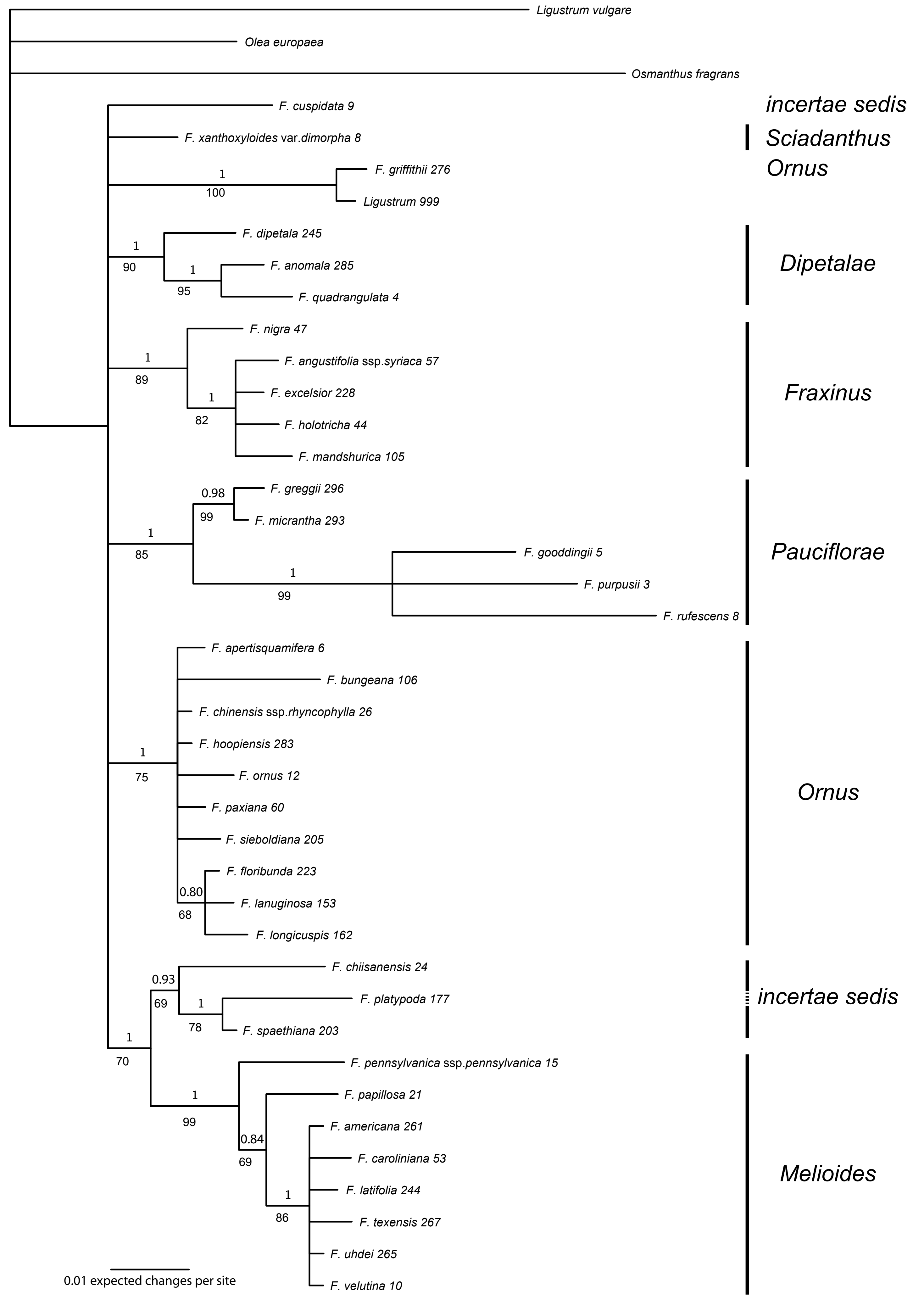

Ligustrum vulgare
Olea europaea
Osmanthus fragrans
F. cuspidata 9
incertae sedis
F. xanthoxyloides var.dimorpha 8
Sciadanthus
1
100
F. griffithii 276
Ligustrum 999
Ornus
F. dipetala 245
1
90
1
95
F. anomala 285
F. quadrangulata 4
Dipetalae
F. nigra 47
1
89
F. angustifolia ssp.syriaca 57
F. excelsior 228
1
82
F. holotricha 44
F. mandshurica 105
Fraxinus
0.98
99
F. greggii 296
F. micrantha 293
1
85
F. gooddingii 5
1
99
F. purpusii 3
F. rufescens 8
Pauciflorae
F. apertisquamifera 6
F. bungeana 106
F. chinensis ssp.rhyncophylla 26
F. hoopiensis 283
1
75
F. ornus 12
F. paxiana 60
F. sieboldiana 205
F. floribunda 223
0.80
68
F. lanuginosa 153
F. longicuspis 162
Ornus
F. chiisanensis 24
0.93
69
F. platypoda 177
1
78
F. spaethiana 203
incertae sedis
1
70
F. pennsylvanica ssp.pennsylvanica 15
1
99
F. papillosa 21
F. americana 261
0.84
69
F. caroliniana 53
F. latifolia 244
1
86
F. texensis 267
F. uhdei 265
F. velutina 10
Melioides
0.01 expected changes per site
