# Supplementary material for: The Phylogeny and Biogeographic History of Ashes (Fraxinus, Oleaceae) Highlight the Roles of Migration and Vicariance in the Diversification of Temperate Trees
Source: PLoS One. 2013 Nov 21;8(11):e80431. doi: 10.1371/journal.pone.0080431 (PMC3837005; doi:10.1371/journal.pone.0080431)
Supplement: Table S1 — Fraxinus samples used in this study, herbarium vouchers and newly published DNA sequences. ID stands for identifier; Sample type: Origin of the sample used in this study; A, Arboretum; W, Collected in the wild; H, Herbarium. Vouchers are deposited at the National Herbarium, Muséum National d'Histoire Naturelle, Paris, France (P00729547 to P00729694), or at the Mexico Herbarium (MEXU1032796 to MEXU991880); Arb.: Arboretum. (DOC) [file pone.0080431.s010.doc]

| Taxa | | Voucher ID | Sample type | Bot. Gard. ID | Sample ID | Country | Origin | Genbank Accession number | | | | |
| --- | --- | --- | --- | --- | --- | --- | --- | --- | --- | --- | --- | --- |
|  |  |  |  |  |  |  |  | *trnH-psbA* | *rpl32-trnL* | ETS | *Phantastica* | ITS |
| *Fraxinus* | *americana* | P00729688 | A | 15 | 2 |  | Arb. National des Barres (Nogent-sur-Vernisson, France) | HM367363 | HM222718 | HQ705329 | HM242426 | HQ705201 |
| *Fraxinus* | *americana* |  | A | 1137 | 35 |  | Arb. National des Barres (Nogent-sur-Vernisson, France) | HM367364 | HM222719 | HQ705330 |  | HQ705203 |
| *Fraxinus* | *americana* | P00729667 | A |  | 117 |  | Pépinière Adeline (La Chapelle Montlinard, France) | HM367362 | HM222717 | HQ705328 | HM242425 | HQ705200 |
| *Fraxinus* | *americana* 'Automn purple' | P00729638 | A | 2793 Kipling Street | 261 | USA | Canopy Association (Palo Alto, U.S.A.) | HM367360 | HM222716 | HQ705327 | HM242423 |  |
| *Fraxinus* | *americana* 'Automn purple' | P00729636 | A | 598 Loma Verde Avenue | 262 | USA | Canopy Association (Palo Alto, U.S.A.) | HM367361 |  |  | HM242424 | HQ705202 |
| *Fraxinus* | *americana* var. *biltmoreana* |  | A | 3826 | 45 |  | Arb. National des Barres (Nogent-sur-Vernisson, France) |  | HM222720 | HQ705331 |  | HQ705204 |
| *Fraxinus* | *angustifolia* | P00729660 | A | 1877 | 17 |  | Arb. National des Barres (Nogent-sur-Vernisson, France) | HM367373 | HM222730 | HQ705342 | HM242437 |  |
| *Fraxinus* | *angustifolia* |  | A | 1929 | 20 |  | Arb. National des Barres (Nogent-sur-Vernisson, France) | HM367374 | HM222731 | HQ705343 | HM242438 | HQ705283 |
| *Fraxinus* | *angustifolia* | P00729574 | A | 1987.1215*A | 214 |  | Sir Harold Hillier Bot. Garden (Romsey, U.K.) | HM367366 | HM222723 | HQ705334 | HM242429 | HQ705205 |
| *Fraxinus* | *angustifolia* | P00729572 | A | 1991.0384*A | 215 | Iran | Sir Harold Hillier Bot. Garden (Romsey, U.K.) | HM367365 | HM222721 | HQ705332 | HM242427 | HQ705207 |
| *Fraxinus* | *angustifolia* ssp. *oxycarpa* |  | A | 1731-1991 | 251 |  | Jardin botanique de Montréal (Montréal, Canada) | HM367367 | HM222724 | HQ705335 | HM242430 | HQ705206 |
| *Fraxinus* | *angustifolia* var. *monophylla* |  | A | 1979.0263*A | 233 |  | Sir Harold Hillier Bot. Garden (Romsey, U.K.) |  | HM222722 | HQ705333 | HM242428 |  |
| *Fraxinus* | *anomala* |  | A | B0702 | 284 | USA | Parc Botanique du Launay (Orsay, France) | HM367379 | HM222738 | HQ705352 | HM242447 | HQ705208 |
| *Fraxinus* | *anomala* |  | A | B0703 | 285 | USA | Parc Botanique du Launay (Orsay, France) | HM367380 | HM222739 | HQ705353 | HM242448 | HQ705209 |
| *Fraxinus* | *apertisquamifera* | P00729616 | W | 1 | aperti1 | Japan | leg Kazuya Iizuka (Utsunomiya University, Japan) | HM367381 | HM222740 | HQ705354 | HM242449 |  |
| *Fraxinus* | *apertisquamifera* | P00729614 | W | 2 | aperti2 | Japan | leg Kazuya Iizuka (Utsunomiya University, Japan) | HM367382 | HM222741 | HQ705355 | HM242450 |  |
| *Fraxinus* | *apertisquamifera* | P00729612 | W | 3 | aperti3 | Japan | leg Kazuya Iizuka (Utsunomiya University, Japan) | HM367383 | HM222742 | HQ705356 | HM242451 |  |
| *Fraxinus* | *apertisquamifera* | P00729610 | W | 4 | aperti4 | Japan | leg Kazuya Iizuka (Utsunomiya University, Japan) | HM367384 | HM222743 | HQ705357 | HM242452 |  |
| *Fraxinus* | *apertisquamifera* | P00729608 | W | 5 | aperti5 | Japan | leg Kazuya Iizuka (Utsunomiya University, Japan) | HM367385 | HM222744 | HQ705358 | HM242453 |  |
| *Fraxinus* | *apertisquamifera* | P00729606 | W | 6 | aperti6 | Japan | leg Kazuya Iizuka (Utsunomiya University, Japan) | HM367386 | HM222745 | HQ705359 | HM242454 |  |
| *Fraxinus* | *apertisquamifera* | P00729602 | W | 8 | aperti8 | Japan | leg Kazuya Iizuka (Utsunomiya University, Japan) | HM367387 | HM222746 | HQ705360 | HM242455 |  |
| *Fraxinus* | *apertisquamifera* | P00729600 | W | 9 | aperti9 | Japan | leg Kazuya Iizuka (Utsunomiya University, Japan) | HM367388 | HM222747 | HQ705361 | HM242456 |  |
| *Fraxinus* | *berlandieriana* | P00729670 | A | 1638 | 14 |  | Arb. National des Barres (Nogent-sur-Vernisson, France) |  | HM222748 | HQ705362 |  |  |
| *Fraxinus* | *berlandieriana* | P00729666 | A | 3617 | 48 |  | Arb. National des Barres (Nogent-sur-Vernisson, France) |  | HM222751 | HQ705366 |  | HQ705211 |
| *Fraxinus* | *berlandieriana* | MEXU991880 | H | 991880 | 19 | Mexico | Mexico Herbarium (Mexico, Mexico) | HM367390 | HM222749 | HQ705364 | HM242457 |  |
| *Fraxinus* | *berlandieriana* | MEXU1158611 | H | 1158611 | 17 | Mexico | Mexico Herbarium (Mexico, Mexico) | HM367389 |  | HQ705363 |  |  |
| *Fraxinus* | *berlandieriana* | P00729567 | A | 1977.1152*Y | 225 |  | Sir Harold Hillier Bot. Garden (Romsey, U.K.) | HM367391 | HM222750 | HQ705365 | HM242458 | HQ705210 |
| *Fraxinus* | *biltmoreana* |  | A | BAYRT - 24302 | 121 |  | Arb. Bayreuth (Bayreuth, Deutschland) | HM367393 |  | HQ705368 | HM242460 |  |
| *Fraxinus* | *biltmoreana* | P00729626 | A |  | 101 |  | Pépinière Adeline (La Chapelle Montlinard, France) | HM367392 | HM222752 | HQ705367 | HM242459 |  |
| *Fraxinus* | *biltmoreana* | P00729569 | A | 1977.2370*Q | 226 |  | Sir Harold Hillier Bot. Garden (Romsey, U.K.) |  | HM222753 | HQ705369 | HM242461 |  |
| *Fraxinus* | *bungeana* | P00729671 | A |  | 69 |  | Arb. Chèvreloup (Rocquencourt, France) | HM367396 | HM222756 | HQ705372 | HM242463 | HQ705213 |
| *Fraxinus* | *bungeana* | P00729682 | A | 3811 | 41 |  | Arb. National des Barres (Nogent-sur-Vernisson, France) | HM367395 | HM222755 | HQ705371 |  |  |
| *Fraxinus* | *bungeana* | P00729618 | A |  | 106 |  | Pépinière Adeline (La Chapelle Montlinard, France) | HM367394 | HM222754 | HQ705370 | HM242462 | HQ705212 |
| *Fraxinus* | *caroliniana* | P00729639 | A |  | 53 |  | Arb. Chèvreloup (Rocquencourt, France) | HM367398 | HM222758 | HQ705374 | HM242465 | HQ705215 |
| *Fraxinus* | *caroliniana* |  | A | 1980.0206*A | 202 | USA | Sir Harold Hillier Bot. Garden (Romsey, U.K.) | HM367397 | HM222757 | HQ705373 | HM242464 | HQ705214 |
| *Fraxinus* | *chiisanensis* |  | A | 8907 | 24 |  | Arb. National des Barres (Nogent-sur-Vernisson, France) | HM367401 | HM222761 | HQ705377 | HM242468 | HQ705218 |
| *Fraxinus* | *chiisanensis* | P00729662 | A | 8994 | 21 |  | Arb. National des Barres (Nogent-sur-Vernisson, France) | HM367399 | HM222759 | HQ705375 | HM242466 | HQ705216 |
| *Fraxinus* | *chiisanensis* | P00729575 | A | 2006.0536*A | 230 |  | Sir Harold Hillier Bot. Garden (Romsey, U.K.) | HM367400 | HM222760 | HQ705376 | HM242467 | HQ705217 |
| *Fraxinus* | *chinensis* | P00729641 | A | 1992 | 31 |  | Arb. National des Barres (Nogent-sur-Vernisson, France) | HM367405 | HM222765 | HQ705381 | HM242472 | HQ705225 |
| *Fraxinus* | *chinensis* | P00729649 | A |  | 108 |  | Pépinière Adeline (La Chapelle Montlinard, France) | HM367402 | HM222762 | HQ705378 | HM242469 | HQ705219 |
| *Fraxinus* | *chinensis* |  | A | 1982.2663*A | 210 |  | Sir Harold Hillier Bot. Garden (Romsey, U.K.) | HM367403 | HM222763 | HQ705379 | HM242470 | HQ705221 |
| *Fraxinus* | *chinensis* | P00729578 | A | 1986.2754*A | 219 |  | Sir Harold Hillier Bot. Garden (Romsey, U.K.) | HM367404 | HM222764 | HQ705380 | HM242471 | HQ705222 |
| *Fraxinus* | *chinensis* ssp. *rhyncophylla* | P00729588 | A | 2140 | 26 |  | Arb. National des Barres (Nogent-sur-Vernisson, France) | HM367408 | HM222768 | HQ705384 | HM242475 | HQ705224 |
| *Fraxinus* | *chinensis* ssp. *rhyncophylla* | P00729656 | A | 3815 | 42 |  | Arb. National des Barres (Nogent-sur-Vernisson, France) | HM367409 | HM222769 | HQ705385 | HM242476 | HQ705226 |
| *Fraxinus* | *chinensis* ssp. *rhyncophylla* | P00729651 | A |  | 109 |  | Pépinière Adeline (La Chapelle Montlinard, France) | HM367406 | HM222766 | HQ705382 | HM242473 | HQ705220 |
| *Fraxinus* | *chinensis* ssp. *rhyncophylla* | P00729577 | A | 2001.0366*A | 229 | South Korea | Sir Harold Hillier Bot. Garden (Romsey, U.K.) | HM367407 | HM222767 | HQ705383 | HM242474 | HQ705223 |
| *Fraxinus* | *cuspidata* | MEXU658827 | H | 658827 | 9 | Mexico | Mexico Herbarium (Mexico, Mexico) | HM367414 | HM222774 | HQ705390 | HM242480 |  |
| *Fraxinus* | *cuspidata* | MEXU758471 | H | 758471 | 2 | Mexico | Mexico Herbarium (Mexico, Mexico) | HM367410 | HM222770 |  |  |  |
| *Fraxinus* | *cuspidata* | MEXU955683 | H | 955683 | 10 | Mexico | Mexico Herbarium (Mexico, Mexico) |  |  | HQ705386 | HM242477 |  |
| *Fraxinus* | *cuspidata* |  | A |  | 290 | USA | Parc Botanique du Launay (Orsay, France) | HM367413 | HM222773 | HQ705389 | HM242479 |  |
| *Fraxinus* | *cuspidata* | P00729601 | A |  | 288 | USA | Peckerwood Garden (Hempstead, U.S.A.) | HM367411 | HM222771 | HQ705387 |  |  |
| *Fraxinus* | *cuspidata* | P00729603 | A |  | 289 | USA | Peckerwood Garden (Hempstead, U.S.A.) | HM367412 | HM222772 | HQ705388 | HM242478 |  |
| *Fraxinus* | *dipetala* | P00729633 | A |  | 56 |  | Arb. Chèvreloup (Rocquencourt, France) |  | HM222777 | HQ705391 | HM242483 | HQ705227 |
| *Fraxinus* | *dipetala* | P00729621 | A |  | 65 |  | Arb. Chèvreloup (Rocquencourt, France) | HM367416 | HM222778 |  | HM242484 |  |
| *Fraxinus* | *dipetala* | P00729560 | A | 10303*A6 | 245 | USA | Rancho Santa Anna Bot. Garden (Claremont, U.S.A.) | HM367415 | HM222776 |  | HM242482 |  |
| *Fraxinus* | *dipetala* | P00729548 | A | 13476*D2 | 243 | USA | Rancho Santa Anna Bot. Garden (Claremont, U.S.A.) |  | HM222775 |  | HM242481 |  |
| *Fraxinus* | *excelsior* |  | A |  | TF | France | Parc Botanique du Launay (Orsay, France) | HM367419 |  |  |  |  |
| *Fraxinus* | *excelsior* | P00729583 | A | 1977.0646 | 232 |  | Sir Harold Hillier Bot. Garden (Romsey, U.K.) | HM367418 |  | HQ705393 |  |  |
| *Fraxinus* | *excelsior* | P00729561 | A | 1977.2024*W | 228 |  | Sir Harold Hillier Bot. Garden (Romsey, U.K.) | HM367417 | HM222779 | HQ705392 | HM242485 |  |
| *Fraxinus* | *excelsior monophylla* | P00729664 | A | 3263 | 50 |  | Arb. National des Barres (Nogent-sur-Vernisson, France) | HM367421 | HM222781 | HQ705395 | HM242487 | HQ705228 |
| *Fraxinus* | *excelsior monophylla* | P00729576 | A | 1982.5733*A | 221 |  | Sir Harold Hillier Bot. Garden (Romsey, U.K.) | HM367420 | HM222780 | HQ705394 | HM242486 |  |
| *Fraxinus* | *excelsior* var. *nana* |  | A | 3337 | 49 |  | Arb. National des Barres (Nogent-sur-Vernisson, France) | HM367422 | HM222782 | HQ705396 |  |  |
| *Fraxinus* | *excelsior* var. *pendula* | P00729652 | A | 350 | 11 |  | Arb. National des Barres (Nogent-sur-Vernisson, France) | HM367423 | HM222783 | HQ705397 | HM242488 | HQ705229 |
| *Fraxinus* | *floribunda* | P00729680 | A | 1739 | 16 |  | Arb. National des Barres (Nogent-sur-Vernisson, France) |  | HM222869 | HQ705534 | HM242591 | HQ705282 |
| *Fraxinus* | *floribunda* | P00729645 | A | 2162 | 27 |  | Arb. National des Barres (Nogent-sur-Vernisson, France) | HM367535 | HM222870 | HQ705536 | HM242593 | HQ705281 |
| *Fraxinus* | *floribunda* | P00729571 | A | 1977.0702*Y | 223 |  | Sir Harold Hillier Bot. Garden (Romsey, U.K.) | HM367425 | HM222785 | HQ705399 | HM242490 | HQ705248 |
| *Fraxinus* | *floribunda* | P00729586 | A | 1977.0718*S | 222 |  | Sir Harold Hillier Bot. Garden (Romsey, U.K.) | HM367424 | HM222784 | HQ705398 | HM242489 | HQ705247 |
| *Fraxinus* | *formosana* | P00729591 | A | OSN84-00-0285-60 | 248 |  | Botanic Garden Osnabrück (Osnabrück, Deutschland) | HM367426 |  | HQ705400 | HM242491 |  |
| *Fraxinus* | *gooddingii* | MEXU1214941 | H | 1214941 | 5 | Mexico | Mexico Herbarium (Mexico, Mexico) | HM367427 | HM222786 | HQ705401 | HM242492 |  |
| *Fraxinus* | *greggii* |  | A | 19860253 | 246 |  | Cambridge University Botanic Garden (Cambridge, U.K.) | HM367429 | HM222787 | HQ705404 | HM242495 | HQ705231 |
| *Fraxinus* | *greggii* | MEXU529898 | H | 529898 | 14 | Mexico | Mexico Herbarium (Mexico, Mexico) | HM367428 |  | HQ705403 | HM242494 |  |
| *Fraxinus* | *greggii* | MEXU779176 | H | 779176 | 13 | Mexico | Mexico Herbarium (Mexico, Mexico) |  |  |  | HM242493 |  |
| *Fraxinus* | *greggii* | MEXU1153309 | H | 1153309 | 12 | Mexico | Mexico Herbarium (Mexico, Mexico) |  |  | HQ705402 |  |  |
| *Fraxinus* | *greggii* | P00729605 | A |  | 294 | USA | Peckerwood Garden (Hempstead, U.S.A.) | HM367431 | HM222789 | HQ705406 |  |  |
| *Fraxinus* | *greggii* | P00729607 | A |  | 296 | USA | Peckerwood Garden (Hempstead, U.S.A.) | HM367433 |  | HQ705408 | HM242498 |  |
| *Fraxinus* | *greggii* | P00729609 | A |  | 295 | USA | Peckerwood Garden (Hempstead, U.S.A.) | HM367432 | HM222790 | HQ705407 | HM242497 |  |
| *Fraxinus* | *greggii* | P00729693 | A | 801 | 281 | USA | University of Arizona Campus Arboretum (Tucson, U.S.A.) | HM367430 | HM222788 | HQ705405 | HM242496 | HQ705230 |
| *Fraxinus* | *griffithii* |  | A | 783-01 | 276 | Japan | Parc Botanique du Launay (Orsay, France) | HM367435 | HM222792 | HQ705410 | HM242500 |  |
| *Fraxinus* | *griffithii* |  | A | 783-03 | 278 | Japan | Parc Botanique du Launay (Orsay, France) |  |  | HQ705412 | HM242502 |  |
| *Fraxinus* | *griffithii* |  | A | 783-10 | 277 | Japan | Parc Botanique du Launay (Orsay, France) | HM367436 |  | HQ705411 | HM242501 |  |
| *Fraxinus* | *griffithii* | P00729581 | A | 1977.2517*T | 234 |  | Sir Harold Hillier Bot. Garden (Romsey, U.K.) | HM367434 | HM222791 | HQ705409 | HM242499 | HQ705232 |
| *Fraxinus* | *guilinensis* S.K.Lee & F.N.Wei | P00729675 | A |  | 68 |  | Arb. Chèvreloup (Rocquencourt, France) | HM367437 | HM222793 | HQ705413 | HM242503 | HQ705233 |
| *Fraxinus* | *holotricha* | P00729668 | A | 3819 | 43 |  | Arb. National des Barres (Nogent-sur-Vernisson, France) | HM367440 | HM222795 | HQ705416 | HM242506 | HQ705236 |
| *Fraxinus* | *holotricha* |  | A | 3820 | 44 |  | Arb. National des Barres (Nogent-sur-Vernisson, France) | HM367441 | HM222796 | HQ705417 | HM242507 | HQ705237 |
| *Fraxinus* | *holotricha* | P00729628 | A |  | 102 |  | Pépinière Adeline (La Chapelle Montlinard, France) | HM367438 | HM222794 | HQ705414 | HM242504 | HQ705234 |
| *Fraxinus* | *holotricha* |  | A | 1982.0066*A | 209 |  | Sir Harold Hillier Bot. Garden (Romsey, U.K.) | HM367439 |  | HQ705415 | HM242505 | HQ705235 |
| *Fraxinus* | *hoopiensis* |  | A | 19990031 | 283 |  | Arboretum Kalmthout (Kalmthout, Belgium) |  | HM222797 |  | HM242508 |  |
| *Fraxinus* | *incana* L. |  | A | BAYRT - 24201 | 122 |  | Arb. Bayreuth (Bayreuth, Deutschland) | HM367442 |  | HQ705419 |  |  |
| *Fraxinus* | *lanuginosa* | P00729691 | A |  | 272 |  | Colvos Creek Nursery (Seattle, U.S.A.) |  |  | HQ705427 | HM242512 |  |
| *Fraxinus* | *lanuginosa* |  | W | 107 | 137 | Japan | Forestry and Forest Products Research Institute (Tsukuba, Japan) | HM367443 | HM222798 | HQ705420 |  |  |
| *Fraxinus* | *lanuginosa* |  | W | 107 | 164 | Japan | Forestry and Forest Products Research Institute (Tsukuba, Japan) | HM367445 | HM222799 | HQ705422 |  |  |
| *Fraxinus* | *lanuginosa* |  | W | 108 | 138 | Japan | Forestry and Forest Products Research Institute (Tsukuba, Japan) | HM367444 |  | HQ705421 | HM242509 |  |
| *Fraxinus* | *lanuginosa* |  | W | 108 | 165 | Japan | Forestry and Forest Products Research Institute (Tsukuba, Japan) | HM367446 |  | HQ705423 | HM242510 |  |
| *Fraxinus* | *lanuginosa* |  | W | 274 | 188 | Japan | Forestry and Forest Products Research Institute (Tsukuba, Japan) | HM367447 |  | HQ705424 |  |  |
| *Fraxinus* | *lanuginosa* |  | W | 275 | 189 | Japan | Forestry and Forest Products Research Institute (Tsukuba, Japan) | HM367448 |  | HQ705425 |  |  |
| *Fraxinus* | *lanuginosa* |  | W | 276 | 190 | Japan | Forestry and Forest Products Research Institute (Tsukuba, Japan) | HM367449 |  | HQ705426 | HM242511 |  |
| *Fraxinus* | *lanuginosa* var. *serrata* |  | W | 52 | 131 | Japan | Forestry and Forest Products Research Institute (Tsukuba, Japan) |  |  | HQ705428 |  |  |
| *Fraxinus* | *lanuginosa* var. *serrata* |  | W | 55 | 132 | Japan | Forestry and Forest Products Research Institute (Tsukuba, Japan) |  |  | HQ705429 |  |  |
| *Fraxinus* | *lanuginosa* var. *serrata* |  | W | 60 | 133 | Japan | Forestry and Forest Products Research Institute (Tsukuba, Japan) |  |  | HQ705430 |  |  |
| *Fraxinus* | *lanuginosa* var. *serrata* |  | W | 78 | 134 | Japan | Forestry and Forest Products Research Institute (Tsukuba, Japan) | HM367450 | HM222800 | HQ705431 |  |  |
| *Fraxinus* | *lanuginosa* var. *serrata* |  | W | 83 | 135 | Japan | Forestry and Forest Products Research Institute (Tsukuba, Japan) | HM367451 | HM222801 | HQ705432 |  |  |
| *Fraxinus* | *lanuginosa* var. *serrata* |  | W | 97 | 136 | Japan | Forestry and Forest Products Research Institute (Tsukuba, Japan) | HM367452 | HM222802 | HQ705433 |  |  |
| *Fraxinus* | *lanuginosa* var. *serrata* |  | W | 113 | 139 | Japan | Forestry and Forest Products Research Institute (Tsukuba, Japan) | HM367453 | HM222803 | HQ705434 |  |  |
| *Fraxinus* | *lanuginosa* var. *serrata* |  | W | 132 | 140 | Japan | Forestry and Forest Products Research Institute (Tsukuba, Japan) | HM367454 | HM222804 | HQ705435 | HM242513 |  |
| *Fraxinus* | *lanuginosa* var. *serrata* |  | W | 139 | 141 | Japan | Forestry and Forest Products Research Institute (Tsukuba, Japan) | HM367455 | HM222805 | HQ705436 | HM242514 |  |
| *Fraxinus* | *lanuginosa* var. *serrata* |  | W | 165 | 142 | Japan | Forestry and Forest Products Research Institute (Tsukuba, Japan) | HM367456 | HM222806 | HQ705437 | HM242515 |  |
| *Fraxinus* | *lanuginosa* var. *serrata* |  | W | 170 | 143 | Japan | Forestry and Forest Products Research Institute (Tsukuba, Japan) | HM367457 | HM222807 | HQ705438 | HM242516 |  |
| *Fraxinus* | *lanuginosa* var. *serrata* |  | W | 176 | 144 | Japan | Forestry and Forest Products Research Institute (Tsukuba, Japan) | HM367458 | HM222808 | HQ705439 |  |  |
| *Fraxinus* | *lanuginosa* var. *serrata* |  | W | 181 | 145 | Japan | Forestry and Forest Products Research Institute (Tsukuba, Japan) | HM367459 | HM222809 | HQ705440 |  |  |
| *Fraxinus* | *lanuginosa* var. *serrata* |  | W | 185 | 146 | Japan | Forestry and Forest Products Research Institute (Tsukuba, Japan) | HM367460 | HM222810 | HQ705441 | HM242517 |  |
| *Fraxinus* | *lanuginosa* var. *serrata* |  | W | 188 | 147 | Japan | Forestry and Forest Products Research Institute (Tsukuba, Japan) | HM367461 | HM222811 | HQ705442 | HM242518 |  |
| *Fraxinus* | *lanuginosa* var. *serrata* |  | W | 191 | 148 | Japan | Forestry and Forest Products Research Institute (Tsukuba, Japan) | HM367462 | HM222812 | HQ705443 |  |  |
| *Fraxinus* | *lanuginosa* var. *serrata* |  | W | 192 | 149 | Japan | Forestry and Forest Products Research Institute (Tsukuba, Japan) | HM367463 | HM222813 | HQ705444 | HM242519 |  |
| *Fraxinus* | *lanuginosa* var. *serrata* |  | W | 194 | 150 | Japan | Forestry and Forest Products Research Institute (Tsukuba, Japan) | HM367464 | HM222814 | HQ705445 | HM242520 |  |
| *Fraxinus* | *lanuginosa* var. *serrata* |  | W | 218 | 152 | Japan | Forestry and Forest Products Research Institute (Tsukuba, Japan) | HM367465 | HM222815 | HQ705446 | HM242521 |  |
| *Fraxinus* | *lanuginosa* var. *serrata* |  | W | 238 | 153 | Japan | Forestry and Forest Products Research Institute (Tsukuba, Japan) | HM367466 | HM222816 | HQ705447 | HM242522 |  |
| *Fraxinus* | *latifolia* | P00729619 | A |  | 66 |  | Arb. Chèvreloup (Rocquencourt, France) | HM367471 | HM222822 | HQ705453 | HM242528 | HQ705243 |
| *Fraxinus* | *latifolia* |  | A | 408 | 3 |  | Arb. National des Barres (Nogent-sur-Vernisson, France) | HM367469 | HM222820 | HQ705451 | HM242526 | HQ705241 |
| *Fraxinus* | *latifolia* |  | A | 3808 | 40 |  | Arb. National des Barres (Nogent-sur-Vernisson, France) | HM367470 | HM222821 | HQ705452 | HM242527 | HQ705242 |
| *Fraxinus* | *latifolia* | P00729615 | A | 10501*C1 | 237 | USA | Rancho Santa Anna Bot. Garden (Claremont, U.S.A.) | HM367467 | HM222817 | HQ705448 | HM242523 | HQ705238 |
| *Fraxinus* | *latifolia* | P00729554 | A | 10521*A3 | 244 | USA | Rancho Santa Anna Bot. Garden (Claremont, U.S.A.) | HM367468 | HM222819 | HQ705450 | HM242525 | HQ705240 |
| *Fraxinus* | *latifolia* | P00729552 | A | 8669*A4 | 238 | USA | Rancho Santa Anna Bot. Garden (Claremont, U.S.A.) |  | HM222818 | HQ705449 | HM242524 | HQ705239 |
| *Fraxinus* | *longicuspis* |  | A | BAYRT - 22607 | 123 |  | Arb. Bayreuth (Bayreuth, Deutschland) | HM367473 |  | HQ705454 |  |  |
| *Fraxinus* | *longicuspis* | P00729690 | A | 2083 | 25 |  | Arb. National des Barres (Nogent-sur-Vernisson, France) | HM367483 | HM222830 | HQ705465 | HM242536 | HQ705245 |
| *Fraxinus* | *longicuspis* |  | W | 1 | 154 | Japan | Forestry and Forest Products Research Institute (Tsukuba, Japan) | HM367474 | HM222824 | HQ705455 |  |  |
| *Fraxinus* | *longicuspis* |  | W | 2 | 155 | Japan | Forestry and Forest Products Research Institute (Tsukuba, Japan) |  |  | HQ705456 | HM242530 |  |
| *Fraxinus* | *longicuspis* |  | W | 3 | 156 | Japan | Forestry and Forest Products Research Institute (Tsukuba, Japan) | HM367475 | HM222825 | HQ705457 | HM242531 |  |
| *Fraxinus* | *longicuspis* |  | W | 4 | 157 | Japan | Forestry and Forest Products Research Institute (Tsukuba, Japan) | HM367476 |  | HQ705458 |  |  |
| *Fraxinus* | *longicuspis* |  | W | 5 | 158 | Japan | Forestry and Forest Products Research Institute (Tsukuba, Japan) | HM367477 | HM222826 | HQ705459 |  |  |
| *Fraxinus* | *longicuspis* |  | W | 6 | 159 | Japan | Forestry and Forest Products Research Institute (Tsukuba, Japan) | HM367478 | HM222827 | HQ705460 | HM242532 |  |
| *Fraxinus* | *longicuspis* |  | W | 7 | 160 | Japan | Forestry and Forest Products Research Institute (Tsukuba, Japan) | HM367479 | HM222828 | HQ705461 |  |  |
| *Fraxinus* | *longicuspis* |  | W | 8 | 161 | Japan | Forestry and Forest Products Research Institute (Tsukuba, Japan) | HM367480 |  | HQ705462 | HM242533 |  |
| *Fraxinus* | *longicuspis* |  | W | 9 | 162 | Japan | Forestry and Forest Products Research Institute (Tsukuba, Japan) | HM367481 | HM222829 | HQ705463 | HM242534 |  |
| *Fraxinus* | *longicuspis* |  | W | 10 | 163 | Japan | Forestry and Forest Products Research Institute (Tsukuba, Japan) | HM367482 |  | HQ705464 | HM242535 |  |
| *Fraxinus* | *longicuspis* | P00729653 | A |  | 110 |  | Pépinière Adeline (La Chapelle Montlinard, France) | HM367472 | HM222823 |  | HM242529 | HQ705244 |
| *Fraxinus* | *longicuspis* var. *latifolia* |  | A | 1438-1972 | 256 |  | Jardin botanique de Montréal (Montréal, Canada) | HM367484 | HM222831 | HQ705466 | HM242537 | HQ705246 |
| *Fraxinus* | *malacophylla* | P00729640 | A |  | 273 |  | Colvos Creek Nursery (Seattle, U.S.A.) | HM367486 |  |  | HM242539 |  |
| *Fraxinus* | *malacophylla* | P00729564 | A | 1969-1384-P | 264 |  | Los Angeles County Arboretum and Botanic Garden (Los Angeles, U.S.A.) | HM367485 |  | HQ705467 | HM242538 |  |
| *Fraxinus* | *mandshurica* | P00729677 | A | 2456 | 22 |  | Arb. National des Barres (Nogent-sur-Vernisson, France) | HM367489 | HM222834 | HQ705470 |  | HQ705251 |
| *Fraxinus* | *mandshurica* | P00729590 | A | 2500 | 23 |  | Arb. National des Barres (Nogent-sur-Vernisson, France) | HM367490 | HM222835 |  |  |  |
| *Fraxinus* | *mandshurica* | P00729620 | A |  | 105 |  | Pépinière Adeline (La Chapelle Montlinard, France) | HM367487 | HM222832 | HQ705468 | HM242540 | HQ705249 |
| *Fraxinus* | *mandshurica* | P00729559 | A | 1982.2662*A | 212 |  | Sir Harold Hillier Bot. Garden (Romsey, U.K.) | HM367488 | HM222833 | HQ705469 | HM242541 | HQ705250 |
| *Fraxinus* | *mandshurica* var. *japonica* |  | W | 263 | 184 | Japan | Forestry and Forest Products Research Institute (Tsukuba, Japan) | HM367491 |  | HQ705471 | HM242542 |  |
| *Fraxinus* | *mandshurica* var. *japonica* |  | W | 264 | 185 | Japan | Forestry and Forest Products Research Institute (Tsukuba, Japan) | HM367492 |  | HQ705472 | HM242543 |  |
| *Fraxinus* | *mandshurica* var. *japonica* |  | W | 265 | 186 | Japan | Forestry and Forest Products Research Institute (Tsukuba, Japan) | HM367493 |  | HQ705473 | HM242544 |  |
| *Fraxinus* | *mandshurica* var. *japonica* |  | W | 296 | 192 | Japan | Forestry and Forest Products Research Institute (Tsukuba, Japan) | HM367494 |  | HQ705474 | HM242545 |  |
| *Fraxinus* | *mandshurica* var. *japonica* |  | W | 297 | 193 | Japan | Forestry and Forest Products Research Institute (Tsukuba, Japan) | HM367495 |  | HQ705475 | HM242546 |  |
| *Fraxinus* | *mandshurica* var. *japonica* |  | W | 298 | 194 | Japan | Forestry and Forest Products Research Institute (Tsukuba, Japan) |  |  | HQ705476 | HM242547 |  |
| *Fraxinus* | *mandshurica* var. *japonica* |  | W | 299 | 195 | Japan | Forestry and Forest Products Research Institute (Tsukuba, Japan) |  |  | HQ705477 |  |  |
| *Fraxinus* | *mariesii* |  | A | BAYRT - 23895 | 124 |  | Arb. Bayreuth (Bayreuth, Deutschland) |  |  | HQ705478 |  | HQ705252 |
| *Fraxinus* | *mariesii* | P00729679 | A | 1883 | 18 |  | Arb. National des Barres (Nogent-sur-Vernisson, France) |  | HM222836 | HQ705479 |  | HQ705253 |
| *Fraxinus* | *micrantha* |  | A |  | 292 |  | Parc Botanique du Launay (Orsay, France) |  | HM222837 | HQ705480 | HM242548 |  |
| *Fraxinus* | *micrantha* |  | A |  | 293 |  | Parc Botanique du Launay (Orsay, France) | HM367496 | HM222838 | HQ705481 | HM242549 |  |
| *Fraxinus* | *nigra* | P00729686 | A | 1994 | 33 |  | Arb. National des Barres (Nogent-sur-Vernisson, France) | HM367498 | HM222840 |  |  |  |
| *Fraxinus* | *nigra* | P00729632 | A | 3828 | 47 |  | Arb. National des Barres (Nogent-sur-Vernisson, France) |  |  | HQ705483 | HM242551 |  |
| *Fraxinus* | *nigra* |  | A |  | 100 |  | Pépinière Adeline (La Chapelle Montlinard, France) | HM367497 | HM222839 | HQ705482 | HM242550 | HQ705254 |
| *Fraxinus* | *obliqua* | P00729547 | A | 1982.3212*A | 207 |  | Sir Harold Hillier Bot. Garden (Romsey, U.K.) | HM367499 | HM222841 | HQ705484 | HM242552 | HQ705255 |
| *Fraxinus* | *ornus* | P00729672 | A |  | 58 |  | Arb. Chèvreloup (Rocquencourt, France) | HM367505 | HM222845 | HQ705490 | HM242560 | HQ705261 |
| *Fraxinus* | *ornus* |  | A | 741 | 1 |  | Arb. National des Barres (Nogent-sur-Vernisson, France) |  | HM222842 | HQ705485 | HM242553 | HQ705256 |
| *Fraxinus* | *ornus* |  | A | 1597 | 12 |  | Arb. National des Barres (Nogent-sur-Vernisson, France) | HM367500 | HM222843 | HQ705486 | HM242554 | HQ705257 |
| *Fraxinus* | *ornus* |  | A | 1887 | 19 |  | Arb. National des Barres (Nogent-sur-Vernisson, France) | HM367501 | HM222844 | HQ705487 | HM242555 | HQ705258 |
| *Fraxinus* | *ornus* | P00729644 | A |  | 274 |  | Colvos Creek Nursery (Seattle, U.S.A.) | HM367502 |  |  | HM242557 | HQ705259 |
| *Fraxinus* | *ornus* |  | A | 1896-1991 | 258 |  | Jardin botanique de Montréal (Montréal, Canada) |  |  | HQ705488 |  |  |
| *Fraxinus* | *ornus* |  | W | MOSOR1 | 286 | Croatia | Leg Yakovlev | HM367503 |  | HQ705489 | HM242558 | HQ705260 |
| *Fraxinus* | *ornus* |  | W | MOSOR2 | 287 | Croatia | Leg Yakovlev | HM367504 |  |  | HM242559 |  |
| *Fraxinus* | *ornus* |  | W |  | BalkansA1 | Croatia | Leg Yakovlev |  |  | HQ705491 |  |  |
| *Fraxinus* | *ornus* |  | W |  | BalkansA2 | Croatia | Leg Yakovlev |  |  | HQ705492 |  |  |
| *Fraxinus* | *ornus* |  | W |  | BalkansA3 | Croatia | Leg Yakovlev |  |  | HQ705493 |  |  |
| *Fraxinus* | *ornus* |  | W |  | BalkansA4 | Croatia | Leg Yakovlev |  |  | HQ705494 |  |  |
| *Fraxinus* | *ornus* |  | W |  | SonjaB1 | Croatia | Leg Yakovlev |  |  | HQ705495 |  |  |
| *Fraxinus* | *ornus* |  | W |  | SonjaB2 | Croatia | Leg Yakovlev |  |  | HQ705496 |  |  |
| *Fraxinus* | *ornus* |  | W |  | SonjaB4 | Croatia | Leg Yakovlev |  |  | HQ705497 |  |  |
| *Fraxinus* | *ornus* |  | W |  | SonjaB5 | Croatia | Leg Yakovlev |  |  | HQ705498 |  |  |
| *Fraxinus* | *oxycarpa* |  | A | BOCH - 0000-RE0-1535 | 270 |  | Botanic Garden Bochum (Bochum, Deutschland) |  |  | HQ705336 | HM242431 | HQ705262 |
| *Fraxinus* | *pallisiae* |  | A | 1034-1976 | 252 |  | Jardin botanique de Montréal (Montréal, Canada) | HM367371 | HM222728 | HQ705340 | HM242435 |  |
| *Fraxinus* | *pallisiae* | P00729624 | A |  | 103 |  | Pépinière Adeline (La Chapelle Montlinard, France) | HM367368 | HM222725 | HQ705337 | HM242432 | HQ705263 |
| *Fraxinus* | *pallisiae* | P00729570 | A | 1982.0951*A | 217 |  | Sir Harold Hillier Bot. Garden (Romsey, U.K.) | HM367369 | HM222726 | HQ705338 | HM242433 | HQ705264 |
| *Fraxinus* | *pallisiae* | P00729584 | A | 1982.0951*B | 218 |  | Sir Harold Hillier Bot. Garden (Romsey, U.K.) | HM367370 | HM222727 | HQ705339 | HM242434 | HQ705265 |
| *Fraxinus* | *papillosa* | MEXU714886 | H | 714886 | 22 | Mexico | Mexico Herbarium (Mexico, Mexico) |  |  | HQ705501 |  |  |
| *Fraxinus* | *papillosa* | MEXU1193510 | H | 1193510 | 1 | Mexico | Mexico Herbarium (Mexico, Mexico) |  |  | HQ705499 | HM242561 |  |
| *Fraxinus* | *papillosa* | MEXU1205968 | H | 1205968 | 21 | Mexico | Mexico Herbarium (Mexico, Mexico) |  |  | HQ705500 | HM242562 |  |
| *Fraxinus* | *papillosa* | P00729692 | A | 1541 | 280 | USA | University of Arizona Campus Arboretum (Tucson, U.S.A.) | HM367507 | HM222847 | HQ705502 | HM242563 | HQ705266 |
| *Fraxinus* | *paxiana* | P00729629 | A |  | 59 |  | Arb. Chèvreloup (Rocquencourt, France) | HM367509 | HM222849 | HQ705504 | HM242565 | HQ705268 |
| *Fraxinus* | *paxiana* | P00729556 | A |  | 60 |  | Arb. Chèvreloup (Rocquencourt, France) | HM367510 | HM222850 | HQ705505 | HM242566 | HQ705269 |
| *Fraxinus* | *paxiana* | P00729657 | A |  | 112 |  | Pépinière Adeline (La Chapelle Montlinard, France) | HM367508 | HM222848 | HQ705503 | HM242564 | HQ705267 |
| *Fraxinus* | *pennsylvanica* |  | W |  | StranShepelyn3 | Ukraine | Leg Yakovlev |  |  | HQ705506 |  |  |
| *Fraxinus* | *pennsylvanica* |  | W |  | StranShepelyn4 | Ukraine | Leg Yakovlev |  |  | HQ705507 |  |  |
| *Fraxinus* | *pennsylvanica* | P00729622 | A |  | 104 |  | Pépinière Adeline (La Chapelle Montlinard, France) | HM367511 | HM222851 | HQ705508 | HM242567 | HQ705270 |
| *Fraxinus* | *pennsylvanica* ssp. *pennsylvanica* | P00729648 | A | 1609 | 13 |  | Arb. National des Barres (Nogent-sur-Vernisson, France) |  | HM222852 | HQ705509 | HM242568 |  |
| *Fraxinus* | *pennsylvanica* ssp. *pennsylvanica* | P00729678 | A | 1639 | 15 |  | Arb. National des Barres (Nogent-sur-Vernisson, France) |  | HM222853 | HQ705510 | HM242569 | HQ705271 |
| *Fraxinus* | *pennsylvanica* ssp. *pennsylvanica* | | A | 3806 | 38 |  | Arb. National des Barres (Nogent-sur-Vernisson, France) | HM367512 | HM222854 | HQ705511 |  |  |
| *Fraxinus* | *platypoda* | P00729635 | A |  | 55 |  | Arb. Chèvreloup (Rocquencourt, France) | HM367524 | HM222860 | HQ705524 | HM242581 | HQ705274 |
| *Fraxinus* | *platypoda* |  | W | 253 | 174 | Japan | Forestry and Forest Products Research Institute (Tsukuba, Japan) | HM367514 | HM222856 | HQ705513 | HM242571 |  |
| *Fraxinus* | *platypoda* |  | W | 254 | 175 | Japan | Forestry and Forest Products Research Institute (Tsukuba, Japan) | HM367515 | HM222857 | HQ705514 | HM242572 |  |
| *Fraxinus* | *platypoda* |  | W | 255 | 176 | Japan | Forestry and Forest Products Research Institute (Tsukuba, Japan) | HM367516 | HM222858 | HQ705515 | HM242573 |  |
| *Fraxinus* | *platypoda* |  | W | 256 | 177 | Japan | Forestry and Forest Products Research Institute (Tsukuba, Japan) | HM367517 |  | HQ705516 | HM242574 |  |
| *Fraxinus* | *platypoda* |  | W | 257 | 178 | Japan | Forestry and Forest Products Research Institute (Tsukuba, Japan) | HM367518 |  | HQ705517 | HM242575 |  |
| *Fraxinus* | *platypoda* |  | W | 258 | 179 | Japan | Forestry and Forest Products Research Institute (Tsukuba, Japan) | HM367519 |  | HQ705518 | HM242576 |  |
| *Fraxinus* | *platypoda* |  | W | 259 | 180 | Japan | Forestry and Forest Products Research Institute (Tsukuba, Japan) | HM367520 |  | HQ705519 | HM242577 |  |
| *Fraxinus* | *platypoda* |  | W | 260 | 181 | Japan | Forestry and Forest Products Research Institute (Tsukuba, Japan) | HM367521 |  | HQ705520 | HM242578 |  |
| *Fraxinus* | *platypoda* |  | W | 261 | 182 | Japan | Forestry and Forest Products Research Institute (Tsukuba, Japan) | HM367522 |  | HQ705521 |  |  |
| *Fraxinus* | *platypoda* |  | W | 262 | 183 | Japan | Forestry and Forest Products Research Institute (Tsukuba, Japan) | HM367523 |  | HQ705522 | HM242579 |  |
| *Fraxinus* | *platypoda* |  | A | 19810042 | 250 |  | Jard. Bot. Nat. de Belgique (Meise, Belgium) |  | HM222859 | HQ705523 | HM242580 | HQ705273 |
| *Fraxinus* | *platypoda* | P00729647 | A |  | 107 |  | Pépinière Adeline (La Chapelle Montlinard, France) | HM367513 | HM222855 | HQ705512 | HM242570 | HQ705272 |
| *Fraxinus* | *potamophila* | P00729674 | A |  | 52 |  | Arb. Chèvreloup (Rocquencourt, France) | HM367372 | HM222729 | HQ705341 | HM242436 | HQ705275 |
| *Fraxinus* | *profunda* |  | A | BAYRT - 11348 | 125 | USA | Arb. Bayreuth (Bayreuth, Deutschland) | HM367525 | HM222861 | HQ705525 |  | HQ705276 |
| *Fraxinus* | *profunda* |  | A | 2001 | 32 |  | Arb. National des Barres (Nogent-sur-Vernisson, France) | HM367563 | HM222889 | HQ705567 |  |  |
| *Fraxinus* | *profunda* |  | A | 2003 | 30 |  | Arb. National des Barres (Nogent-sur-Vernisson, France) | HM367529 | HM222865 | HQ705529 | HM242585 |  |
| *Fraxinus* | *profunda* |  | A | 5525 | 34 |  | Arb. National des Barres (Nogent-sur-Vernisson, France) | HM367564 | HM222890 | HQ705568 | HM242621 |  |
| *Fraxinus* | *profunda* | P00729593 | A | OSN93-12-0041-10 | 249 |  | Botanic Garden Osnabrück (Osnabrück, Deutschland) | HM367527 | HM222863 | HQ705527 | HM242583 | HQ705278 |
| *Fraxinus* | *profunda* |  | A | 1251-2001 | 254 |  | Jardin botanique de Montréal (Montréal, Canada) | HM367528 | HM222864 | HQ705528 | HM242584 | HQ705279 |
| *Fraxinus* | *profunda* | P00729582 | A | 1993.1156*A | 216 | USA | Sir Harold Hillier Bot. Garden (Romsey, U.K.) | HM367526 | HM222862 | HQ705526 | HM242582 | HQ705277 |
| *Fraxinus* | *purpusii* | MEXU833610 | H | 833610 | 18 | Mexico | Mexico Herbarium (Mexico, Mexico) | HM367530 |  | HQ705531 | HM242586 |  |
| *Fraxinus* | *purpusii* | MEXU904442 | H | 904442 | 3 | Mexico | Mexico Herbarium (Mexico, Mexico) |  |  | HQ705532 | HM242587 |  |
| *Fraxinus* | *purpusii* | MEXU967925 | H | 967925 | 16 | Mexico | Mexico Herbarium (Mexico, Mexico) |  |  | HQ705530 |  |  |
| *Fraxinus* | *quadrangulata* |  | A | 518 | 4 |  | Arb. National des Barres (Nogent-sur-Vernisson, France) | HM367532 | HM222867 |  | HM242589 |  |
| *Fraxinus* | *quadrangulata* |  | A | 519 | 5 |  | Arb. National des Barres (Nogent-sur-Vernisson, France) |  | HM222868 |  | HM242590 |  |
| *Fraxinus* | *quadrangulata* | P00729655 | A |  | 111 |  | Pépinière Adeline (La Chapelle Montlinard, France) | HM367531 | HM222866 |  | HM242588 |  |
| *Fraxinus* | *raibocarpa* |  | A | BAYRT - 24237 | 126 |  | Arb. Bayreuth (Bayreuth, Deutschland) | HM367533 |  | HQ705533 |  |  |
| *Fraxinus* | *retusa* var. *henryana* |  | A |  | 119 |  | Pépinière Adeline (La Chapelle Montlinard, France) | HM367534 |  | HQ705535 | HM242592 | HQ705280 |
| *Fraxinus* | *richardii* |  | A | BAYRT - 23819 | 127 |  | Arb. Bayreuth (Bayreuth, Deutschland) | HM367536 | HM222871 | HQ705537 |  |  |
| *Fraxinus* | *rufescens* | MEXU738146 | H | 738146 | 7 | Mexico | Mexico Herbarium (Mexico, Mexico) |  |  | HQ705538 |  |  |
| *Fraxinus* | *rufescens* | MEXU928691 | H | 928691 | 8 | Mexico | Mexico Herbarium (Mexico, Mexico) |  |  | HQ705539 | HM242594 |  |
| *Fraxinus* | *sieboldiana* |  | W | 145 | 166 | Japan | Forestry and Forest Products Research Institute (Tsukuba, Japan) | HM367538 |  | HQ705541 | HM242596 |  |
| *Fraxinus* | *sieboldiana* |  | W | 146 | 167 | Japan | Forestry and Forest Products Research Institute (Tsukuba, Japan) | HM367539 |  | HQ705542 | HM242597 |  |
| *Fraxinus* | *sieboldiana* |  | W | 147 | 168 | Japan | Forestry and Forest Products Research Institute (Tsukuba, Japan) | HM367540 | HM222873 | HQ705543 | HM242598 |  |
| *Fraxinus* | *sieboldiana* |  | W | 148 | 169 | Japan | Forestry and Forest Products Research Institute (Tsukuba, Japan) | HM367541 |  | HQ705544 | HM242599 |  |
| *Fraxinus* | *sieboldiana* |  | W | 150 | 170 | Japan | Forestry and Forest Products Research Institute (Tsukuba, Japan) | HM367542 |  | HQ705545 | HM242600 |  |
| *Fraxinus* | *sieboldiana* |  | W | 151 | 171 | Japan | Forestry and Forest Products Research Institute (Tsukuba, Japan) | HM367543 |  | HQ705546 | HM242601 |  |
| *Fraxinus* | *sieboldiana* |  | W | 152 | 172 | Japan | Forestry and Forest Products Research Institute (Tsukuba, Japan) | HM367544 | HM222874 | HQ705547 | HM242602 |  |
| *Fraxinus* | *sieboldiana* |  | W | 153 | 173 | Japan | Forestry and Forest Products Research Institute (Tsukuba, Japan) | HM367545 | HM222875 | HQ705548 | HM242603 |  |
| *Fraxinus* | *sieboldiana* |  | W | 272 | 187 | Japan | Forestry and Forest Products Research Institute (Tsukuba, Japan) |  |  | HQ705549 | HM242604 |  |
| *Fraxinus* | *sieboldiana* |  | A | 880-1988 | 253 |  | Jardin botanique de Montréal (Montréal, Canada) | HM367547 | HM222877 |  | HM242606 |  |
| *Fraxinus* | *sieboldiana* | P00729661 | A |  | 114 |  | Pépinière Adeline (La Chapelle Montlinard, France) | HM367537 | HM222872 | HQ705540 | HM242595 | HQ705284 |
| *Fraxinus* | *sieboldiana* | P00729599 | A | 1977.5390*Q | 205 |  | Sir Harold Hillier Bot. Garden (Romsey, U.K.) | HM367546 | HM222876 | HQ705550 | HM242605 | HQ705285 |
| *Fraxinus* | *sikkimensis* |  |  |  | 120 |  | Arb. Frankfürt (Frankfürt, Deutshland) | HM367548 |  | HQ705551 | HM242607 | HQ705286 |
| *Fraxinus* | *sogdiana* |  | A | 1002-2003 | 259 |  | Jardin botanique de Montréal (Montréal, Canada) |  | HM222732 | HQ705345 | HM242440 | HQ705288 |
| *Fraxinus* | *sogdiana* | P00729565 | A | 1977.1184*W | 224 |  | Sir Harold Hillier Bot. Garden (Romsey, U.K.) |  |  | HQ705344 | HM242439 | HQ705287 |
| *Fraxinus* | sp. |  | A |  | 71 |  | Arb. Chèvreloup (Rocquencourt, France) | HM367553 | HM222881 | HQ705554 | HM242612 | HQ705290 |
| *Fraxinus* | sp. |  | A |  | 70 |  | Arb. Chèvreloup (Rocquencourt, France) | HM367552 | HM222880 |  | HM242611 |  |
| *Fraxinus* | sp. |  | W |  | StranShepelyn2 | Ukraine | Leg Yakovlev |  |  | HQ705555 |  |  |
| *Fraxinus* | sp. |  | W |  | StranShepelyn31 | Ukraine | Leg Yakovlev |  |  | HQ705556 |  |  |
| *Fraxinus* | sp. |  | W |  | StranShepelyn41 | Ukraine | Leg Yakovlev |  |  | HQ705557 |  |  |
| *Fraxinus* | sp. | P00729573 | A | 1977.5154 | 235 |  | Sir Harold Hillier Bot. Garden (Romsey, U.K.) | HM367550 | HM222878 | HQ705552 | HM242609 |  |
| *Fraxinus* | sp. | P00729579 | A | 1977.5155 | 236 |  | Sir Harold Hillier Bot. Garden (Romsey, U.K.) | HM367551 | HM222879 | HQ705553 | HM242610 |  |
| *Fraxinus* | sp. | P00729568 | A | 1977.1210*Q | 211 |  | Sir Harold Hillier Bot. Garden (Romsey, U.K.) | HM367549 |  |  | HM242608 | HQ705289 |
| *Fraxinus* | *spaethiana* | P00729658 | A | 3825 | 46 |  | Arb. National des Barres (Nogent-sur-Vernisson, France) | HM367557 | HM222885 | HQ705561 |  |  |
| *Fraxinus* | *spaethiana* | P00729566 | A | 1977.5400*Q | 208 |  | Sir Harold Hillier Bot. Garden (Romsey, U.K.) | HM367556 | HM222884 | HQ705560 | HM242615 |  |
| *Fraxinus* | *spaethiana* | P00729551 | A | 1977.5614*W | 204 |  | Sir Harold Hillier Bot. Garden (Romsey, U.K.) | HM367555 | HM222883 | HQ705559 | HM242614 | HQ705292 |
| *Fraxinus* | *spaethiana* |  | A | 1977.5659*R | 203 |  | Sir Harold Hillier Bot. Garden (Romsey, U.K.) | HM367554 | HM222882 | HQ705558 | HM242613 | HQ705291 |
| *Fraxinus* | *syriaca* |  | A | BAYRT - 23925 | 128 |  | Arb. Bayreuth (Bayreuth, Deutschland) |  |  | HQ705350 | HM242445 | HQ705294 |
| *Fraxinus* | *syriaca* | P00729631 | A |  | 57 |  | Arb. Chèvreloup (Rocquencourt, France) | HM367378 | HM222737 | HQ705351 | HM242446 | HQ705298 |
| *Fraxinus* | *syriaca* |  | A | 2019 | 29 |  | Arb. National des Barres (Nogent-sur-Vernisson, France) | HM367376 | HM222734 | HQ705347 | HM242442 | HQ705296 |
| *Fraxinus* | *syriaca* | P00729643 | A | 2020 | 28 |  | Arb. National des Barres (Nogent-sur-Vernisson, France) | HM367375 | HM222733 | HQ705346 | HM242441 | HQ705295 |
| *Fraxinus* | *syriaca* |  | A | 3804 | 39 |  | Arb. National des Barres (Nogent-sur-Vernisson, France) | HM367377 | HM222735 | HQ705348 | HM242443 | HQ705297 |
| *Fraxinus* | *syriaca* | P00729665 | A |  | 116 |  | Pépinière Adeline (La Chapelle Montlinard, France) |  | HM222736 | HQ705349 | HM242444 | HQ705293 |
| *Fraxinus* | *texensis* | P00729587 | A | 19980148 | 268 | USA | Fort Worth Bot. Garden (Fort Worth, U.S.A.) |  |  | HQ705563 | HM242617 |  |
| *Fraxinus* | *texensis* | P00729597 | A |  | 269 | USA | Fort Worth Bot. Garden (Fort Worth, U.S.A.) | HM367559 | HM222887 | HQ705564 | HM242618 |  |
| *Fraxinus* | *texensis* | P00729589 | A |  | 267 | USA | Fort Worth Bot. Garden (Fort Worth, U.S.A.) | HM367558 | HM222886 | HQ705562 | HM242616 |  |
| *Fraxinus* | *tomentosa* |  | A | BAYRT - 23872 | 129 |  | Arb. Bayreuth (Bayreuth, Deutschland) | HM367561 |  | HQ705565 |  |  |
| *Fraxinus* | *tomentosa* |  | A | 2298-1961 | 255 |  | Jardin botanique de Montréal (Montréal, Canada) | HM367562 | HM222888 | HQ705566 | HM242620 | HQ705300 |
| *Fraxinus* | *tomentosa* | P00729669 | A |  | 118 |  | Pépinière Adeline (La Chapelle Montlinard, France) | HM367560 |  |  | HM242619 | HQ705299 |
| *Fraxinus* | *trifoliata* | P00729611 | A | 14849*B2 | 242 | USA | Rancho Santa Anna Bot. Garden (Claremont, U.S.A.) |  | HM222891 | HQ705570 |  |  |
| *Fraxinus* | *trifoliata* | P00729613 | A | 17232*C2 | 241 | USA | Rancho Santa Anna Bot. Garden (Claremont, U.S.A.) | HM367565 |  | HQ705569 | HM242622 | HQ705301 |
| *Fraxinus* | *turkestanica* |  | A | BAYRT - 24437 | 130 |  | Arb. Bayreuth (Bayreuth, Deutschland) | HM367566 | HM222892 | HQ705571 |  | HQ705302 |
| *Fraxinus* | *uhdei* |  | A | 19890759 | 247 |  | Cambridge University Botanic Garden (Cambridge, U.K.) | HM367567 | HM222893 | HQ705572 | HM242623 | HQ705303 |
| *Fraxinus* | *uhdei* | P00729634 | A | 1636 Edgewood Drive | 263 | USA | Canopy Association (Palo Alto, U.S.A.) | HM367569 | HM222895 | HQ705574 | HM242625 |  |
| *Fraxinus* | *uhdei* | P00729642 | A | 3825 Fabian Way | 260 | USA | Canopy Association (Palo Alto, U.S.A.) | HM367568 | HM222894 | HQ705573 | HM242624 | HQ705304 |
| *Fraxinus* | *uhdei* | P00729646 | A |  | 275 | USA | Colvos Creek Nursery (Seattle, U.S.A) |  | HM222898 | HQ705577 | HM242628 |  |
| *Fraxinus* | *uhdei* | P00729562 | A | 1963-0485-P | 265 |  | Los Angeles County Arboretum and Botanic Garden (Los Angeles, U.S.A.) |  | HM222896 | HQ705575 | HM242626 | HQ705305 |
| *Fraxinus* | *uhdei* | P00729558 | A | 1967-1367-P | 266 |  | Los Angeles County Arboretum and Botanic Garden (Los Angeles, U.S.A.) | HM367570 | HM222897 | HQ705576 | HM242627 |  |
| *Fraxinus* | *velutina* | P00729625 | A |  | 63 |  | Arb. Chèvreloup (Rocquencourt, France) | HM367571 | HM222902 | HQ705581 | HM242630 | HQ705312 |
| *Fraxinus* | *velutina* | P00729684 | A | 219 | 10 |  | Arb. National des Barres (Nogent-sur-Vernisson, France) |  | HM222899 | HQ705578 | HM242629 | HQ705306 |
| *Fraxinus* | *velutina* |  | A | 241 | 7 |  | Arb. National des Barres (Nogent-sur-Vernisson, France) | HM367572 | HM222903 | HQ705582 |  |  |
| *Fraxinus* | *velutina* | P00729650 | A | 242 | 6 |  | Arb. National des Barres (Nogent-sur-Vernisson, France) |  | HM222901 | HQ705580 |  |  |
| *Fraxinus* | *velutina* | P00729663 | A |  | 115 |  | Pépinière Adeline (La Chapelle Montlinard, France) |  | HM222900 | HQ705579 |  | HQ705307 |
| *Fraxinus* | *velutina* var. *coriacea* | P00729623 | A |  | 64 |  | Arb. Chèvreloup (Rocquencourt, France) | HM367575 | HM222906 | HQ705585 | HM242633 | HQ705313 |
| *Fraxinus* | *velutina* var. *coriacea* | P00729550 | A | 15292*C2 | 240 | USA | Rancho Santa Anna Bot. Garden (Claremont, U.S.A.) | HM367574 | HM222905 | HQ705584 | HM242632 | HQ705311 |
| *Fraxinus* | *velutina* var. *coriacea* | P00729687 | A | 5942*B3 | 239 | USA | Rancho Santa Anna Bot. Garden (Claremont, U.S.A.) | HM367573 | HM222904 | HQ705583 | HM242631 | HQ705310 |
| *Fraxinus* | *velutina* var. *glabra* | P00729654 | A | 1149 | 36 |  | Arb. National des Barres (Nogent-sur-Vernisson, France) | HM367576 | HM222907 | HQ705586 |  |  |
| *Fraxinus* | *velutina* var. *glabra* |  | A | 1150 | 37 |  | Arb. National des Barres (Nogent-sur-Vernisson, France) | HM367577 | HM222908 | HQ705587 | HM242635 |  |
| *Fraxinus* | *velutina* var. *glabra* | P00729555 | A | 1977.7018*S | 201 |  | Sir Harold Hillier Bot. Garden (Romsey, U.K.) |  |  |  | HM242634 |  |
| *Fraxinus* | *velutina* var. *toumeyi* | P00729563 | A | 1977.0656*U | 227 |  | Sir Harold Hillier Bot. Garden (Romsey, U.K.) | HM367579 | HM222910 | HQ705589 | HM242637 | HQ705309 |
| *Fraxinus* | *velutina* var. *toumeyi* | P00729549 | A | 1977.5304*W | 206 |  | Sir Harold Hillier Bot. Garden (Romsey, U.K.) | HM367578 | HM222909 | HQ705588 | HM242636 | HQ705308 |
| *Fraxinus* | *xanthoxyloides* | P00729627 | A |  | 61 |  | Arb. Chèvreloup (Rocquencourt, France) |  |  | HQ705592 | HM242640 | HQ705320 |
| *Fraxinus* | *xanthoxyloides* |  | A |  | 62 |  | Arb. Chèvreloup (Rocquencourt, France) | HM367582 | HM222913 |  | HM242641 |  |
| *Fraxinus* | *xanthoxyloides* | P00729659 | A |  | 113 |  | Pépinière Adeline (La Chapelle Montlinard, France) | HM367580 | HM222911 | HQ705590 | HM242638 | HQ705316 |
| *Fraxinus* | *xanthoxyloides* | P00729580 | A | 1986.2709*A | 220 |  | Sir Harold Hillier Bot. Garden (Romsey, U.K.) | HM367581 | HM222912 | HQ705591 | HM242639 | HQ705318 |
| *Fraxinus* | *xanthoxyloides* var. *dimorpha* | P00729694 | A | 229 | 8 |  | Arb. National des Barres (Nogent-sur-Vernisson, France) | HM367584 | HM222915 | HQ705593 | HM242643 | HQ705314 |
| *Fraxinus* | *xanthoxyloides* var. *dimorpha* |  | A | 259 | 9 |  | Arb. National des Barres (Nogent-sur-Vernisson, France) |  | HM222916 | HQ705594 | HM242644 | HQ705315 |
| *Fraxinus* | *xanthoxyloides* var. *dimorpha* | P00729676 | A | 3018 | 51 |  | Arb. National des Barres (Nogent-sur-Vernisson, France) | HM367583 | HM222914 |  | HM242642 | HQ705319 |
| *Fraxinus* | *xanthoxyloides* var. *dumosa* | P00729673 | A |  | 67 |  | Arb. Chèvreloup (Rocquencourt, France) | HM367586 | HM222917 |  | HM242646 | HQ705321 |
| *Fraxinus* | *xanthoxyloides* var. *dumosa* | P00729553 | A | 1978.1290*Q | 200 |  | Sir Harold Hillier Bot. Garden (Romsey, U.K.) | HM367585 |  |  | HM242645 | HQ705317 |
| **Outgroups** | |  |  |  |  |  |  |  |  |  |  |  |
| *Forsythia* | x *intermedia* |  | A |  | 8 |  | Parc Botanique du Launay (Orsay, France) | HM367506 |  | HQ705596 |  |  |
| *Jasminum* | sp. | P00729596 | A | batiment 350 | 995 |  | Parc Botanique du Launay (Orsay, France) |  |  | HQ705597 |  | HQ705323 |
| *Jasminum* | sp. | P00729594 | A | Batiment 360 | 998 |  | Parc Botanique du Launay (Orsay, France) |  |  | HQ705598 |  | HQ705324 |
| *Ligustrum* | *vulgare* |  | A |  | 2 |  | Parc Botanique du Launay (Orsay, France) | HM367587 | HM222920 | HQ705599 | HM242647 |  |
| *Ligustrum* | *vulgare* | P00729617 | W |  | 999 |  | Orsay, France |  |  | HQ705600 | HM242648 | HQ705325 |
| *Olea* | *europea* |  | A |  | 3 |  | Parc Botanique du Launay (Orsay, France) | HM367588 |  |  | HM242649 |  |
| *Osmanthus* | *fragrans* |  | A |  | 4 |  | Parc Botanique du Launay (Orsay, France) | HM367589 | HM222921 | HQ705601 | HM242650 |  |
| *Phillyrea* | *angustifolia* |  | A |  | 1 |  | Parc Botanique du Launay (Orsay, France) | HM367590 | HM222922 | HQ705602 |  |  |
| *Syringa* | *vulgaris* | P00729598 | A |  | 5 |  | Parc Botanique du Launay (Orsay, France) | HM367591 |  | HQ705603 |  |  |
| *Syringa* | *vulgaris* | P00729592 | A |  | 997 |  | Parc Botanique du Launay (Orsay, France) | HM367592 | HM222923 | HQ705604 |  | HQ705326 |
