# Supplementary material for: The Phylogeny and Biogeographic History of Ashes (Fraxinus, Oleaceae) Highlight the Roles of Migration and Vicariance in the Diversification of Temperate Trees
Source: PLoS One. 2013 Nov 21;8(11):e80431. doi: 10.1371/journal.pone.0080431 (PMC3837005; doi:10.1371/journal.pone.0080431)
Supplement: Figure S9 — Phylogenetic tree resulting from the BEAST analysis of the four datasets (cpDNA – trnH-psbA and rpl32-trnL, nETS, nITS and phantastica). Bars at nodes represent the 95% confidence interval; posterior probabilities above the branches. Ages in million years before present. (PDF) [file pone.0080431.s009.pdf]

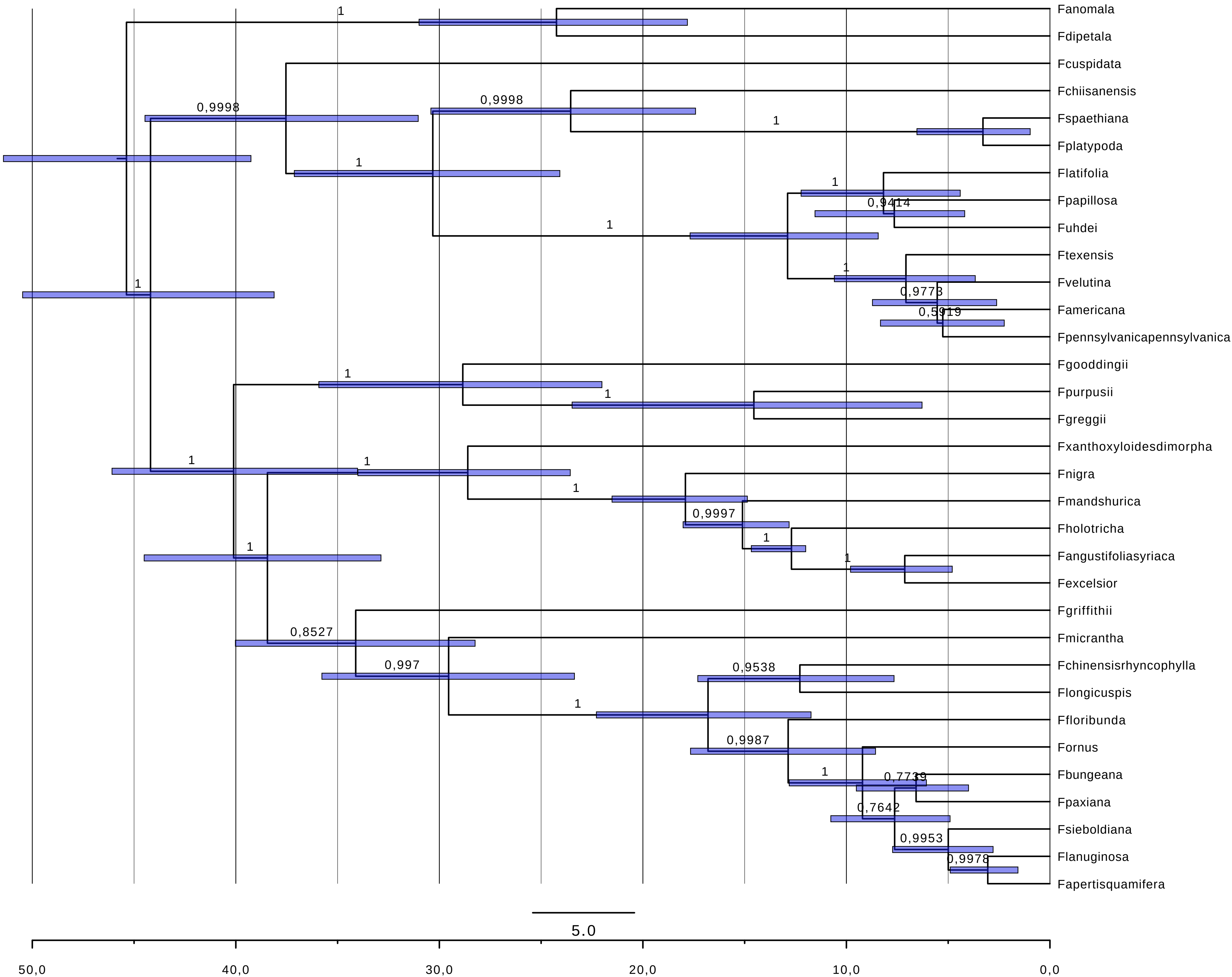
Fanomala
Fdipetala
Fcuspidata
Fchiisanensis
Fspaethiana
Fplatypoda
Flatifolia
Fpapillosa
Fuhdei
Ftexensis
Fvelutina
Famericana
Fpennsylvanicapennsylvanica
Fgooddingii
Fpurpusii
Fgreggii
Fxanthoxyloidesdimorpha
Fnigra
Fmandshurica
Fholotricha
Fangustifoliasyriaca
Fexcelsior
Fgriffithii
Fmicrantha
Fchinensisrhyncophylla
Flongicuspis
Ffloribunda
Fornus
Fbungeana
Fpaxiana
Fsieboldiana
Flanuginosa
Fapertisquamifera
1
0,9998
0,9998
1
1
1
0,9414
1
1
0,9773
0,5919
1
1
1
1
1
0,9997
1
1
1
0,8527
0,997
0,9538
1
0,9987
1
0,7739
0,7642
0,9953
0,9978
5.0
50,0
40,0
30,0
20,0
10,0
0,0
